# Past, Present and Future Perspectives of Forensic Genetics

**DOI:** 10.3390/biom15050713

**Published:** 2025-05-13

**Authors:** Itzae Adonai Gutiérrez-Hurtado, Mayra Elizabeth García-Acéves, Yolanda Puga-Carrillo, Mariano Guardado-Estrada, Denisse Stephania Becerra-Loaiza, Víctor Daniel Carrillo-Rodríguez, Reynaldo Plazola-Zamora, Juliana Marisol Godínez-Rubí, Héctor Rangel-Villalobos, José Alonso Aguilar-Velázquez

**Affiliations:** 1 Departamento de Biología Molecular y Genómica, Centro Universitario de Ciencias de la Salud, Universidad de Guadalajara, Guadalajara 44340, Jalisco, Mexico; 2 Instituto de Investigación en Genética Molecular, Departamento de Ciencias Médicas y de la Vida, Centro Universitario de la Ciénega, Universidad de Guadalajara, Ocotlán 47810, Jalisco, Mexico; 3 Laboratorio de Ciencias Morfológico Forenses y Medicina Molecular, Departamento de Morfología, Centro Universitario de Ciencias de la Salud, Universidad de Guadalajara, Guadalajara 44340, Jalisco, Mexico; 4 Maestría en Genética Forense e Identificación Humana, Centro Universitario de Ciencias de la Salud, Universidad de Guadalajara, Guadalajara 44340, Jalisco, Mexico; 5 Laboratorio de Genética, Escuela Nacional de Ciencias Forenses, Universidad Nacional Autónoma de México, Coyoacán 04510, Ciudad de Mexico, Mexico; 6 Departamento de Aparatos y Sistemas II, Universidad Autónoma de Guadalajara, Zapopan 45129, Jalisco, Mexico; 7 Departamento de Morfología, Centro Universitario de Ciencias de la Salud, Universidad de Guadalajara, Guadalajara 44340, Jalisco, Mexico

**Keywords:** forensic genetics, molecular markers, STR, SNP, INDEL, massive parallel sequencing, mitochondrial DNA

## Abstract

Forensic genetics has experienced remarkable advancements over the past decades, evolving from the analysis of a limited number of DNA segments to comprehensive genome-wide investigations. This progression has significantly improved the ability to establish genetic profiles under diverse conditions and scenarios. Beyond individual identification, forensic genetics now enables the inference of physical traits (e.g., eye, hair, and skin color, as well as body composition), biogeographic ancestry, lifestyle habits such as alcohol and tobacco use, and even the transfer of genital microbiomes post-coitus, among other characteristics. Emerging trends point to a future shaped by the integration of cutting-edge technologies, including CRISPR-Cas systems, artificial intelligence, and machine learning, which promise to further revolutionize the field. This review provides a thorough exploration of forensic genetics, tracing its evolution from its foundational methods (past) to its diverse modern applications (present) and offering insights into its potential future directions.

## 1. Introduction

Forensic sciences apply scientific principles and methodologies to analyze evidence collected from crime scenes, ultimately helping identify, detect, examine, and convict criminals. This attractive and emerging field comprises various disciplines, including forensic biology, forensic chemistry, ballistics, digital forensics, forensic anthropology, forensic pathology and forensic genetics, each playing independent yet cooperative roles in criminal investigations and judicial decisions [1].

Forensic genetics uses DNA polymorphisms (known in this field as molecular markers) to identify individuals in the resolution of biological relationships and criminal cases. Over the last ~40 years, this area has been in constant evolution, from the use of large molecular weight markers known as variable numbers of tandem repeats (VNTRs) to the use of small segments of DNA like short tandem repeats (STRs) and single nucleotide polymorphisms (SNPs) that are currently analyzed using sophisticated techniques such as capillary electrophoresis (CE) and, more recently, massively parallel sequencing (MPS) [2,3]. These advances have allowed the possibility of identifying a person more accurately through DNA, determining not only their genetic profile but also inferring their physical characteristics, drug use, and biogeographic ancestry, among many other characteristics [4,5]. Therefore, in this paper, we make an extensive review where we address the past, present, and future of forensic genetics according to current trends.

## 2. The Past of Forensic Genetics

### 2.1. Blood Groups

Thanks to the knowledge provided by Mendel’s laws, elements of biological origin inherited from parents to children began to be used as determinants of paternity, for example, diseases, physical characteristics, psychological traits, pathological and ethnic characteristics, etc. However, in 1900 the ABO system was discovered by Karl Landsteiner [6], which represented the first system of biological markers that followed a Mendelian inheritance, but their application in paternity tests was not until 1910 by von Dungern and Hirszfeld’s demonstration [7]. Subsequently, M and N factors were discovered in 1927 [8,9] and the RH factor in 1940 [10], which represents three systems of blood factors (A-B-O, M-N, and Rh) with 216 varieties of blood demonstrable [11]. These systems worked for more than 50 years because it was an easy methodology (positive or negative reaction), the genetics were well known, and the frequencies of the antigens were available. However, one of the limitations of the application in paternity tests was their limited power of exclusion. Wiener calculated the possibility of exclusion for the A-B-O system at 18%, with the M-N system at 20% and the Rh-Hr system at 25% [11] and the combination of the three systems was a 50.8% chance of exclusion [12,13]. This percentage was increased to 67–69% by the introduction of the Ss antigens and the Kell, Duffy, and Kidd systems, with seven blood group systems [14,15,16,17]. Blood groups represent the first era of paternity testing; however, they were not equally relevant for human identification, and due to their limited power of exclusion, it was necessary to search for other biological markers.

### 2.2. Human Leukocyte Antigen (HLA)

In 1958 the first antigen on white blood cells (WBCs) was discovered. This antigen is part of a system known as the Human Leukocyte Antigen (HLA) and was studied primarily for its association with solid organ transplant survival [18]. HLA is the major histocompatibility system in humans and has a great diversity of antigens and *loci*. In paternity cases, the most used *loci* were HLA-A and HLA-B, with codominant expression and two antigens for locus, which are inherited in pairs, forming haplotypes [19]. Many antigen combinations and low frequency in the population result in a high prior probability of exclusion (PPE), with around 90% or greater [19,20,21,22]. This PPE was much higher than the 67–69% offered by the extended ABO system (ABO, Rh, MNS, Duffy, Kidd and Kell) and, combined with the blood group, has a PPE of over 95–97.2%. The methodology used for HLA typing was based on the microlymphocytotoxicity test, where the lymphocytes were combined with a panel of specific antibodies. Subsequently, the antigen–antibody reaction was added with the complement, and the cytotoxic effect was visualized by staining [19]. This methodology was available for many laboratories, and the results were reliable and reproducible. Although the HLA system was a powerful tool for disputed paternity, it had the same limitations as blood groups, and it was necessary to use other markers to confirm paternity; for example, in cases with HLA combinations of antigens that confirm paternity, paternity was then excluded by ABO systems [21,23,24,25]; these results were expected in around 5–6% of all cases [25].

During the early 1990s, commercial kits such as the PM (Polymarker) and DQA1 (DQ alpha) systems were widely implemented for forensic human identification and paternity testing. These kits, developed by Roche and Applied Biosystems (Waltham, MA, USA), were based on the amplification of specific DNA regions followed by allele detection through reverse dot blot hybridization using allele-specific oligonucleotide probes. The PM kit included five additional loci (LDLR, GYPA, HBGG, D7S8, and GC), while DQA1 targeted the highly polymorphic HLA-DQA1 locus. These systems offered several advantages, including compatibility with partially degraded samples and relatively fast processing times for that period. However, as PCR multiplexing technology advanced and PCR-based kits became more widely available, the use of PM + DQA1 systems gradually declined by the late 1990s [26].

### 2.3. Serum Proteins

In the same decade as the discovery of the HLA system, genetic differences in serum protein haptoglobin [27] were reported. Subsequently, other serum proteins and erythrocyte isoenzyme markers with desirable characteristics in paternity tests (Mendelian inheritance and population variation) were described [28]. Some of the proteins and enzymes that were most used for this purpose were the following: group-specific component (Gc), transferrin (Tf), haptoglobin (Hp), ceruloplasmin (Cp) and erythrocyte enzymes, such as phosphoglucomutase (PGM), acid phosphatase (acP), adenylate kinase (AK), adenosine deaminase (ADA), and 6-phosphogluconate dehydrogenase (6-PGD) [29,30,31,32,33,34,35,36].

The methodology for visualizing the phenotype of proteins and enzymes was through electrophoresis to separate allelic products with different molecular weights [27]. The support medium depended on the protein or enzyme (polyacrylamide, agarose, or starch), but one of the most used was acrylamide, using different techniques like McCombs and Bowman [33]. In addition, in later years, genetic polymorphisms of seven human lymphocyte proteins analyzed by two-dimensional electrophoresis were included, with the advantage that all the polymorphisms could be seen in the same gel and with independent transmission [37].

Exclusions of paternity were direct or indirect; direct exclusion was when a genetic marker was present in the child and was absent in the mother and alleged father; indirect exclusion is when there is an absence of an expected marker in the child when the alleged father is presumed homozygous [32]. The total exclusion rate observed was 62% for serum proteins [28] and 67% for human lymphocyte proteins [37]. Nevertheless, serum proteins were less used than in other systems, but they were used as a complement or to corroborate inconclusive results with other systems like HLA or blood groups [28]. Finally, although these three systems could increase the power of exclusion to more than 90%, this was still insufficient to resolve all cases and even more complex cases such as incest [38]. In addition, they were still not sufficiently relevant for human identification cases in forensic applications.

### 2.4. DNA Fingerprinting: Variable Number of Tandem Repeats

In the mid-1980s, Professor Alec Jeffreys was interested in studying genetic variation among individuals and conducted some early research to detect genetic differences in humans. However, the breakthrough did not come from his initial project of interest but rather from an unrelated study involving the analysis of the myoglobin gene in seal meat [39,40]. When he and his colleagues compared the myoglobin gene in seals to the human counterpart, they discovered that some short repetitive sequences were homologous between the two species. Upon further comparison with published sequences of tandem repeats known as minisatellites, they found these sequences to be identical [41]. Professor Jeffreys realized these repeating sequences “could be highly variable, informative genetic markers” [42]. His group developed a radioactive probe, composed of short sequences, which could bind to these minisatellite sequences and ultimately reveal patterns unique to every individual: a DNA “fingerprint” [43].

Minisatellites, also known as variable numbers of tandem repeats (VNTRs), provide highly polymorphic, individual-specific DNA genetic fingerprints [42,43,44]. VNTRs are non-coding tandem sequence repeats ranging from 10 to 100 nucleotides in length. It is estimated that there are approximately 1500 VNTRs in the human genome, but they are often clustered near the telomere, or end of the chromosome [45,46]. These units can be repeated two to several hundred times at each minisatellite. Their use as markers for linkage studies began in the early 1980s, when they were employed to map chromosomal regions associated with various Mendelian diseases. However, after the discovery of DNA fingerprinting, the findings gained widespread attention through media coverage, and Professor Jeffreys was invited to participate in several legal cases. First, in an immigration dispute involving a family from Ghana, DNA fingerprinting confirmed for the first time the relationship between a mother and her son [47], resolving doubts about his identity and paving the way for its use in forensic cases and identity verification. Later, in the Colin Pitchfork case, DNA profiling linked the rape and murder of two girls to a single perpetrator and exonerated an initial suspect, marking the first time DNA cleared someone of a crime. A mass DNA screening of local men eventually led to the apprehension of Colin Pitchfork, whose DNA matched the crime scene evidence, securing his conviction [39]. This successful outcome established DNA profiling as a valuable tool in solving crimes. Since then, VNTR polymorphisms have been used in genetic association studies and as genetic markers in paternity testing and forensic investigations. Later, DNA typing was refined by introducing a new technique: the polymerase chain reaction (PCR). DNA amplification by PCR provides increased sensitivity, thus allowing small amounts of DNA to be analyzed, even from archival and partially degraded samples. However, some years later they were practically forgotten due to their technical limitations for forensic applications, such as low number in the genome, chromosomal location (mainly at the chromosome ends), and the large size of DNA fragments required for the analysis, which limits the profiling of degraded or extremely limited DNA samples. Therefore, the introduction of other markers helped to solve the limitations of the VNTRs, such as microsatellites, also called short tandem repeats (STRs), as well as the single nucleotide polymorphisms (SNPs) and insertion/deletions (INDELs), which, thanks to their smaller size, make them easier to detect in samples with a low amount of DNA [39].

## 3. The Present of Forensic Genetics

### 3.1. STRs: The Gold Standard Markers in Forensic Genetics

**Figure 1:**
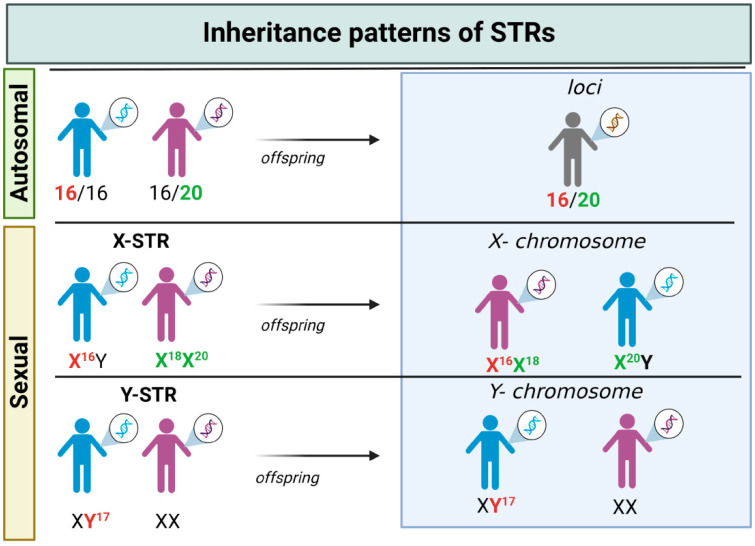
Inheritance patterns of STR markers. For autosomal STRs, the genotype consists of one allele inherited at random from each parent. In contrast, the inheritance patterns of sex chromosome STRs differ based on the type of chromosome. For X-STRs, males inherit a single allele exclusively from their mother due to the presence of only one X chromosome per cell. In females, the X-STR genotype comprises one allele inherited from the father and one from the mother, resembling the inheritance pattern observed in autosomal STRs (due to the presence of two X chromosomes). Lastly, Y-STRs are present only in males under normal conditions. Each male individual possesses a single allele for each Y-STR, inherited exclusively through the paternal line.

STRs were described as a prominent genetic marker for human identity in the early 1990s. They were structurally analogous to minisatellites but with shorter repeat tracts, thus easier to amplify and multiplex. Some of the characteristics that made STRs the genetic markers of choice in forensic practice [48] were the following: (I) are located in non-coding regions, only about 8% are located in coding regions (mainly in intronic regions) [49]; (II) abundance in the genome [49,50], on the average, one STRs occurs per 2000 pb; (III) highly polymorphic DNA sequences (heterozygosity >70%) with repeat units of 2–6 pb (the most popular are 4 bp), alleles are named by the number of the repeat sequence on the STR locus (i.e., Allele 4: GATA-GATA-GATA-GATA) [51], and they are present both in autosomal chromosomes (aSTRs) and in sexual chromosomes (Y-STRs in the Y chromosome and X-STRs in the X chromosomes) presenting different inheritance patterns (Figure 1); (IV) can vary in size from person to person (discrimination power >0.8) without impacting the health [52]. Thanks to these characteristics, the Forensic Science Service (FSS) began an exhaustive search for new STR candidates [53]. With the prominent success of identifying new STRs for forensic application, the FBI (Federal Bureau of Investigation) Laboratory led U.S. efforts to establish the first STR core *loci* to form CODIS (Combined DNA Index System). This project was launched in April 1996 and selected the following 13 STR loci: CSF1PO, FGA, TH01, TPOX, VWA, D3S1358, D5S818, D7S820, D8S1179, D13S317, D16S539, D18S51, and D21S11 [54]. The STR *locus* is represented by codes composed of letters and numbers, for example, D21S11, where D represents DNA, 21 represents chromosome 21 on which the STR locus is located, S stands for STR, and 11 is the unique identifier [55].

Later, the development of the capillary electrophoresis (CE) fluorescence detection method facilitated the separation and visualization of multiplex PCR fragments: PCR products are separated by size and dye color by electrophoresis, followed by induced fluorescence by laser with multi-wavelength detection [3]. This methodology, based on PCR and capillary electrophoresis (PCR-CE), has been the most used for amplifying STRs and is the current gold standard for human identification in forensic laboratories.

For many years, manufacturers such as Applied Biosystems and Promega Corp. have developed commercial kits that target the 13 CODIS loci and, subsequently, other additional STRs (Table 1). Relevant forensic agencies, SWGDAM (Scientific Working Group on DNA Analysis Methods) and ENFSI (European Network of Forensic Science Institutes) [56,57], require standardization and validation procedures for the implementation of STR typing kits. Additionally, population studies are carried out to evaluate a priori forensic parameters that show the informativeness of a commercial kit in a population before its application in real forensic casework. The number of STRs of the CODIS has been expanded, and presently most HID (human identification) kits include at least 20 autosomal STR loci [58] (Table 1).

A crucial resource in forensic practice is the creation of DNA databases, which have become one of the most efficient tools in criminal investigations for searching for unknown perpetrators. Each country can select a defined number of STR *loci* that must be routinely typed for each sample to be accepted for inclusion in the database. The selection of these STRs may depend on experimental data from collaborative exercises, commercially available kits, and recommendations from the Interpol DNA working group in 1998 [70].

**Figure 2:**
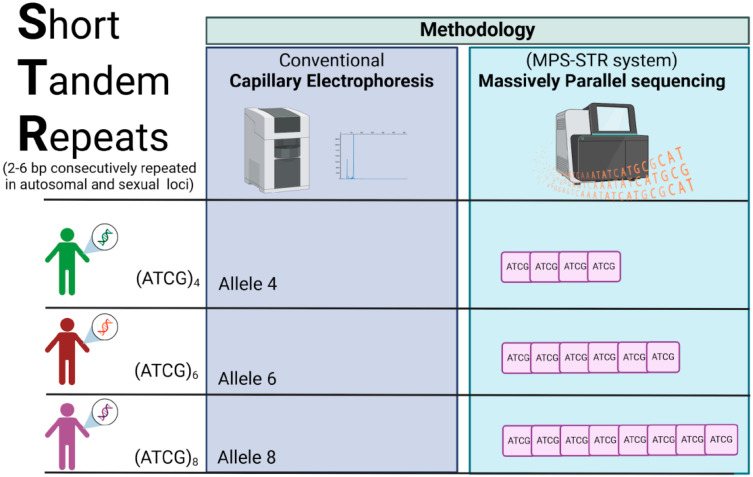
Comparison of the obtained data from the STR analyses based on capillary electrophoresis (CE) and massive parallel sequencing (MPS). Using electropherograms obtained through CE, it is possible to identify STR alleles (e.g., alleles 4, 6, or 8). In contrast, MPS technology allows for the identification of both the allele and its nucleotide sequence, providing information on length-based alleles as well as those based on the sequence of repeats (LB-alleles and RSB-alleles, respectively).

For the last fifteen years, a new massive genotyping technology has emerged: massively parallel sequencing (MPS). MPS allows simultaneous genotyping and sequencing of many different genetic markers, like autosomal STRs (aSTRs), X- and Y-linked STRs (X-STRs and Y-STRs, respectively), and SNPs. In addition to the above, MPS generates more information than PCR-CE technology because it not only allows us to know the length of the alleles (long-based [LB] alleles) but also performs sequencing of the alleles, generating alleles based on the sequence (repeat sequence-based [RSB] alleles), which allows us to detect alleles with the same length but with different internal sequences (Figure 2). This technology allows genomic systems to be more capable of solving both biological relationship tests and criminal cases. Some of the forensic genomic systems studied are the Verogen’s ForenSeq line (ForenSeq DNA Signature Prep, ForenSeq Mitochondrial DNA Control Region, ForenSeq Mitochondrial Complete Genome, and ForenSeq Imagen) was sourced from Verogen Inc. (11111 Flintkote Avenue, San Diego, CA, USA). The PowerSeq® Whole Mito System and PowerSeq® 46GY System kits were obtained from Promega Corporation (2800 Woods Hollow Road, Madison, WI, USA), among others. 

Finally, it is relevant to mention the existence of informative resources with information on the central STRs involved in human identification tests. One of the widely used is the National Institute of Standards and Technology Short Tandem Repeat Internet Database created in 1997, which is commonly referred to as STRBase (http://www.cstl.nist.gov/biotech/strbase/ (accessed on 17 February 2025)) [71], which includes allele variants, triallelic patterns, addresses for scientists working with STRs and databases, and population data summaries. STRBase is a database to compile and consolidate the extensive scientific literature related to STRs. This platform is an essential tool for the standardization and development of DNA analysis, particularly in the forensic field, as it facilitates the typing of genetic profiles. On the other hand, YHRD (Y Chromosome Haplotype Reference Database) is a free-access platform available at https://yhrd.org/. It is a Y-chromosome reference database established in 1999 by the Institute of Legal Medicine and Forensic Sciences at Humboldt University in Berlin. Its purpose is to collect and organize Y chromosome haplotypes from male individuals across various regions of the world. YHRD provides tools for haplotype searches, frequency estimation, and biogeographic relationship analysis through the frequency distribution of Y-STR and Y-SNP markers, made possible by the collaboration of multiple forensic laboratories worldwide [72]. Today, YHRD reports a total of 349,750 haplotypes. Among its main datasets are the following: Y12, with 309,090 haplotypes; Y17, with 289,405 haplotypes; Y23, with 103,280 haplotypes; Y27, with 106,444 haplotypes; and a category that includes all available Y-STR markers in the platform, with 46,773 registered haplotypes. More recently, the STRs for identity ENFSI Reference database (STRidER) V3/R3 was launched to create a functional online, freely accessible forensic platform (available online at https://strider.online, accessed on 18 February 2025) for the database and interpretation and quality control of autosomal STR data [73].

### 3.2. SNPs and INDELs: Complementary Binary Genetic Markers

**Figure 3:**
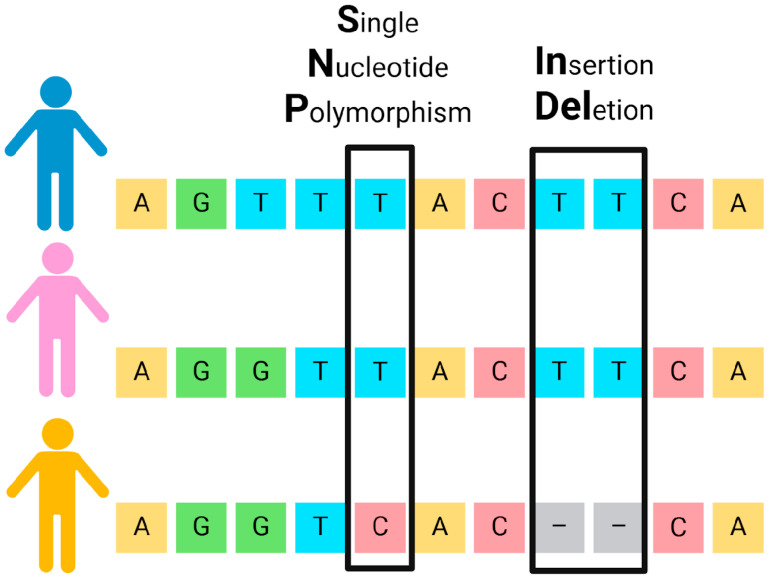
Graphical representation of SNPs and INDELs in three different individuals. While SNPs represent single nucleotide changes, INDELs conform to the presence or absence of a short nucleotide sequence. Each colored box represents a specific nucleotide (A, G, T, or C), and the black rectangles highlight the locations of the variants: a SNP (left) and an INDEL (right). Gray boxes with dashes indicate deleted nucleotides. The three figures on the left represent different individuals.

In recent years, SNPs have become prominent as a genetic marker for human identification. SNPs are point mutations in the DNA sequence that occur throughout the human genome, in both coding and non-coding regions (including autosomal, sex-linked, and mitochondrial DNA) [74]. These mutations involve the substitution of one nucleotide for another [75,76] (Figure 3).

Several characteristics make SNPs more advantageous for forensic casework than STRs: they require smaller amplicon sizes (50–150 bp), are more frequent in the human genome (about 1 in every 1000 bp, totaling millions per individual, making them the most common form of human genetic variation), have a lower mutation rate, and are highly suitable for high-throughput genotyping through multiplexed sequencing [74,76,77,78,79,80]. These features make SNPs particularly useful for analyzing old, degraded, or low-copy biological samples (where DNA fragments may be too short for STR analysis), for kinship and paternity testing (especially when relationships span multiple generations), and for research in population and evolutionary genetics [80,81,82].

Despite these advantages, there are some drawbacks and limitations in using SNPs as primary markers in forensic investigations instead of STRs, at least for now. These include their lower discrimination power (since SNPs are mostly bi-allelic, requiring many loci to match the discriminative power of STRs) and the extensive use of established STR kits and databases in the forensic community worldwide [77].

Currently, the forensic community uses SNPs for various purposes. Based on their applications, SNPs can be categorized into four types [77]: identity informative SNPs (iiSNPs), used for individual differentiation; lineage SNPs, which provide data for kinship/paternity testing and evolutionary studies; ancestry informative SNPs (aiSNPs), used to infer the biogeographical origin of the DNA owner; and phenotype informative SNPs (piSNPs), which predict visible traits like skin, hair, eye color, height, weight, facial features, etc., known as External Visible Characteristics (EVCs) [48].

Among methods for analyzing SNPs, the SNaPshot^®^ mini-sequencing method (Applied Biosystems) is frequently used because it does not require additional equipment beyond what is already present in forensic labs [79]. Other techniques, such as TaqMan^®^ hybridization probes, hybridization microarrays, and massive parallel sequencing (MPS), have been discussed in the literature [79,83,84,85,86]. Depending on specific objectives (e.g., sequencing a single gene, whole-exome sequencing, or whole-genome sequencing), some MPS platforms are more appropriate than others due to their unique characteristics. Although SNPs are primarily bi-allelic markers, MPS can perform multiplexing, allowing for the simultaneous analysis of numerous SNPs [87].

Another type of biallelic genetic marker consists of the insertion/deletion (INDEL) of short nucleotide segments (Figure 3). INDELs share some advantages with both STRs and SNPs: they have short amplicons, typically 60–160 bp, and low mutation rates, making them suitable for the analysis of degraded DNA [88,89]. Regarding SNPs, an advantage is the ease of analysis that does not require additional steps post-PCR before analysis using capillary electrophoresis. Thousands of INDELs have been characterized in the human genome [88,89,90] and selected to produce multiplexes that have been developed for analysis of INDELs. For example, the Investigator DIPplex^®^ Kit (Qiagen GmbH, Hilden, Germany) simultaneously amplifies 30 biallelic *loci* [90].

Although SNPs and INDELs have specific advantages over STRs, the latter remain the gold standard markers in most forensic cases. Consequently, SNPs and INDELs are generally regarded as supplementary markers, useful when STRs provide inconclusive results or fail to produce the expected outcomes. MPS-based forensic kits now incorporate STRs and SNPs, enhancing the informativeness of these genomic systems. This combined approach has already been evaluated in various populations worldwide, demonstrating its effectiveness [91,92,93,94,95,96].

### 3.3. Statistical Analysis and Interpretation of Autosomal Genetic Evidence

In forensic investigations, once a genetic profile is obtained—whether from STRs, SNPs, or INDELs—the next step is to perform a statistical analysis to determine the evidentiary weight of the genetic findings. In cases involving biological relationships, this analysis typically involves the calculation of a value known as the likelihood ratio (LR), also referred to as the paternity, sibship, or grandparentage index, depending on the type of relationship being evaluated. The LR expresses how many times more likely the observed genetic data are under one hypothesis compared to an alternative (e.g., paternity vs. non-paternity). Additionally, the probability of paternity (W) is often calculated, indicating the probability that the alleged relationship is true [97,98,99]. On the other hand, in criminal casework, the random match probability (RMP) is commonly used. This metric estimates the probability that an unrelated individual, selected at random from a given population, would coincidentally share the same genetic profile as the one found at the crime scene. The LR can also be applied in this context, representing how many times the suspect’s genetic profile is more likely to be observed compared to others in the population [100].

To carry out these calculations, a variety of specialized statistical software tools are available, including PatPCR, Familias, FamlinkX, DNAVIEW, PopStats, StatsDNA, DNAStatistX, STRmix, LRmix Studio, and EuroForMix, among others [97]. Furthermore, international organizations and professional societies have published guidelines and best practices that should be followed to ensure the proper implementation and interpretation of these statistical analyses [101,102,103,104].

### 3.4. Mitochondrial DNA Polymorphisms: A Tool of Maternal Heritage

**Figure 4:**
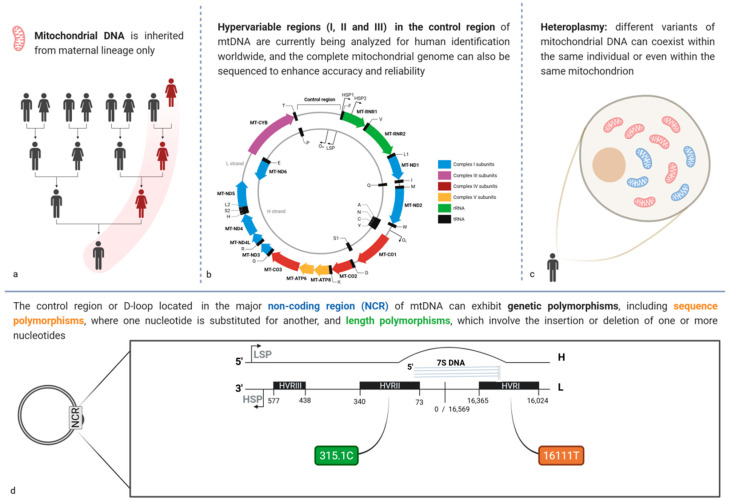
Representation of some key characteristics of the mtDNA: (**a**) it is inherited exclusively through the maternal lineage to all her children; (**b**) the mtDNA presents variations between mitochondria and even in the same mitochondrion; (**c**) it presents three non-coding hypervariable regions (HVI, HVII and HVIII) that are very useful for human identification purposes due to the accumulation of mutations; and (**d**) which can be sequence polymorphisms (change in one nucleotide for another) and length polymorphisms (addition or deletion of nucleotides). In forensic genetics, a specific classification is given to mutations regarding the Cambridge Reference Sequence (CRS); for example, when a G is changed to a C at position 16111, the position of the nucleotide where the change occurred plus the nucleotide that was changed is specified so that the final notation would be 16111C, whereas if a length polymorphism occurs where a C was added at position 315, the final notation would be 315.1C.

Mitochondrial DNA (mtDNA) is a circular, extranuclear, maternally inherited genome of 16,569 bp that has some peculiar characteristics different from nuclear DNA (Figure 4a). MtDNA is located within the inner membrane of mitochondria, and each cell has hundreds to thousands of copies [105]. Additionally, it can be extracted from teeth, bones, and hair shafts, which can be useful in forensic scenarios where soft tissue samples may be scarce or intentionally or naturally highly degraded [106]. Thus, in some cases, analysis of mtDNA polymorphisms can help identify individuals who are victims of massive disasters, war crimes, or terrorist attacks [106,107,108]. However, although these characteristics are attractive for forensic cases, it is important to keep in mind its limitations.

Unlike the analysis of STRs, the analysis of mtDNA for forensic purposes can be a long and laborious process. MtDNA features a control region of 1121 base pairs, which is highly polymorphic. This control region contains three hypervariable regions (HVR), named HVRI (16,024 to 16,365), HVRII (73 to 340), and HVRIII (438 to 574) [106] (Figure 4b). For forensic purposes, the polymorphisms of the HVRI and HVRII, and to a lesser extent the HVRIII regions, are analyzed through Sanger sequencing [109]. However, specific considerations must be applied in the workflow when analyzing mtDNA due to the characteristics of the samples analyzed. Thus, it must work according to an implemented quality management system and the recommendations issued by international organizations such as the International Forensic Society [110,111,112].

An important fact that makes the mtDNA analysis different from other genetic markers is that some cells could have more than one mitochondrial genome, which is present in the form of a “heterozygous state” in some positions. This phenomenon is called heteroplasmy and can be in sequence or length variations (Figure 4c). Therefore, due to their nature, it is important to distinguish between heteroplasmy and sample contamination [113].

Two key factors must be considered when performing casework calculations involving mtDNA. First, the mtDNA population database required to calculate the random match probability (RMP) must include hundreds to thousands of individuals, since this calculation is sensitive to the sample size of the database employed. Moreover, these databases must represent the population where the mtDNA polymorphism analysis is performed. Currently, several population studies analyze the polymorphisms of the hypervariable regions I, II, and even the III region [109,114,115,116,117,118]. The most well-known mtDNA population database is the EMPOP database, released in 2005 [119]. EMPOP incorporates an alignment system that prevents different nomenclatures from being used for the same polymorphism, addressing multiple possible alignments and reducing the risk of erroneous exclusions. Its primary objective is the collection, quality control, and searchable accessibility of mitochondrial DNA haplotypes from various populations worldwide. According to available data, version 14 of EMPOP—the latest online version—contains a total of 63,434 mitotypes, distributed as follows: 63,363 covering the hypervariable region I (HVS-I); 61,272 covering hypervariable regions I and II (HVS-I and HVS-II); 51,813 covering the entire control region (D-loop); and 10,648 including the complete mitogenome.

It would be possible to enhance the discriminatory power of mtDNA analysis by analyzing the entire mitochondrial genome rather than just the control region. This is significant because although the hypervariable regions exhibit a higher rate of polymorphism, they represent only 25% of the total polymorphism of the mtDNA genome [120]. However, sequencing the entire mitochondrial genome using Sanger sequencing cannot be considered a routine task due to the time required to achieve full sequencing. Nevertheless, this challenge has been addressed with the development of MPS technology, which allows the sequencing of the whole genome in a short time and at a lower cost [120]. For instance, sequencing the whole mtDNA genome helps distinguish between two individuals that might share the same HVRI + HVRII regions haplotype, identifying the polymorphisms in the coding region [121]. Finally, although sequencing mtDNA genomes is feasible, a higher sample size of the population database is required to better estimate the discrimination power of this genetic marker.

### 3.5. DNA Phenotyping and Ancestry Inference

Phenotypic traits are influenced by both environmental factors (including diet, exercise, sunlight exposure, and stress) and an individual’s genotype. Therefore, it is possible to predict certain physical traits from a DNA sample. This process is known as Forensic DNA Phenotyping (FDP) [122]. Using a specific selection of SNPs to predict phenotypic information from a DNA sample is one of the most promising applications of SNPs in forensic science [123].

Currently, forensic DNA analysis relies heavily on comparing DNA profiles—biological traces found at crime scenes are analyzed and compared to those of known individuals (suspects) or genetic profiles stored in forensic databases. The new genetic technology, however, aims to extract information about an individual’s phenotypic traits directly from their DNA sample [48,80,82]. This method is particularly useful in cases where there are no potential suspects and no matches between the DNA evidence and profiles in criminal databases. By predicting phenotypic traits from biological samples collected at crime scenes, investigators can gain probabilistic information about the physical characteristics of the DNA donor. This information helps narrow down the list of potential suspects and aids in the investigation. Pigmentation and biogeographic ancestry (BGA) markers often support each other, providing a clearer picture of the individual’s appearance [85].

**Figure 5:**
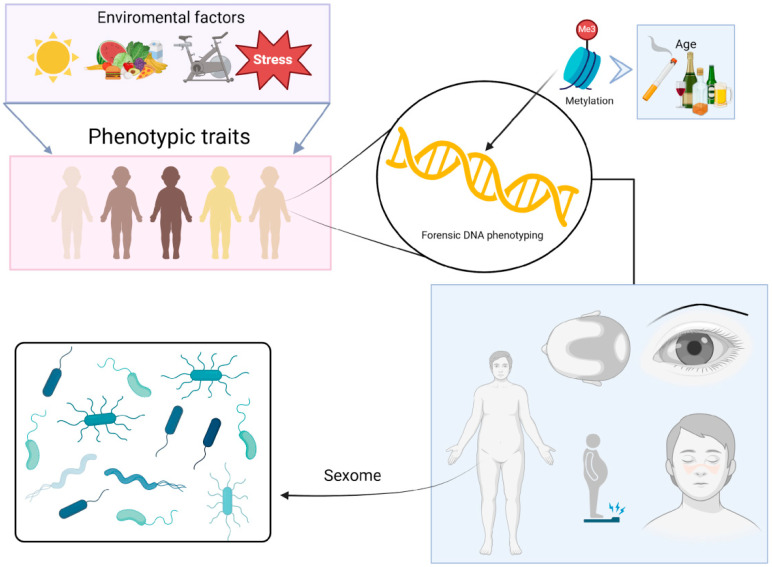
Prediction of physical traits using phenotype-informative markers. While most phenotypes are influenced by environmental factors, certain traits can be inferred from DNA with high accuracy. Examples include hair color, eye color, eyebrow shape, skin pigmentation, height, body build, and freckles, among many others. Additionally, other phenotypes can also be predicted, such as age through methylation patterns and the transfer of the bacterial microbiome after coitus.

A wide range of features has been explored for their potential use in FDP, including (i) predicting iris, hair, and skin color using SNP markers in key genes [124,125]; (ii) determining eyebrow color [126]; (iii) identifying the presence of freckles [127]; (iv) assessing hair shape [128]; (v) detecting male pattern baldness [129]; and (vi) estimating body height [130]. Additionally, other behavioral traits have been inferred, such as tobacco and alcohol consumption habits based on DNA methylation (DNAm) analysis from blood samples [131], and the genetic identification of the genital microbiome exchanged during sexual acts, referred to as the “sexome” by the authors, which may be useful in sexual assault cases [132] (Figure 5).

Another area of significant interest in forensic science is the inference of a person’s age from a biological sample, which can be crucial for distinguishing among potential sample donors. Age inference is particularly important in forensics because the accurate prediction of other traits (such as hair color, body height, and hair loss) directly depends on the age of the sample donor [133]. Numerous studies have been published on age inference, utilizing various methodologies and genetic sources [134,135,136,137,138,139], but epigenetic markers showed the most accurate results [138,139].

Ancestry-Informative Markers (AIMs) are informative genetic markers of ancestry that are used to infer the BGA of an individual. AIMs are characterized by alleles with distinct frequency distributions across populations. In forensic genetics, AIMs are typically short autosomal sequences, such as SNPs, INDELs, or microhaplotypes [133,134,135]. Inferring BGA at a resolution beyond the continental level requires a substantially larger number of markers compared to those used in conventional DNA profiling. High-throughput techniques, such as massively parallel sequencing (MPS), have enabled the analysis of more markers from minimal amounts of DNA [136]. Over the past decade, various commercial and community-developed MPS assays incorporating AIM panels for BGA prediction have become available, including MAPlex [133], the Precision ID Ancestry Panel by Thermo Fisher Scientific (Waltham, MA, USA), the VISAGE Basic Tool [137], and Primer-set B from the ForenSeq™ DNA Signature Prep Kit on the MiSeq FGx system (QIAGEN, Valencia, CA, USA) [138]. Once AIMs are analyzed in a group of individuals or a population, they must be compared to reference data from ancestral populations. This comparison enables the determination of ancestry proportions and interbreeding levels within current populations. Such analysis is particularly valuable in forensic cases involving the identification of individuals from diverse biogeographical origins, such as those affected by mass disasters, migration, or human trafficking cases.

### 3.6. DNA Transfer

DNA analysis techniques have evolved to include highly sensitive tools, making it possible to obtain genetic profiles from very small amounts of DNA (~100 pg, equivalent to 15–20 human cells) recovered at crime scenes [69]. Touch DNA, or transfer DNA evidence, results from human contact with a surface, and the DNA can originate from epithelial cells, cell fragments/nuclei, cell-free DNA, or anucleated corneocytes [140]. Consequently, touch DNA samples typically contain low amounts of DNA.

Currently, transfer DNA analysis is commonly applied in cases of property or violent crimes where no blood or semen traces are present [141]. Several factors can influence the amount of DNA transferred to an object, and these can be grouped into three categories: donor characteristics, surface type, and laboratory methods [142]. DNA donors are often classified as good or poor shedders based on their ability to deposit high or low quantities of DNA on a surface [143]. This shedding ability varies among individuals (e.g., due to age or sex—males tend to shed more DNA than females) and within the same individual under different conditions (e.g., sweating, frequency of handwashing, pressure and friction applied to the surface, contact area—fingertips vs. palms—and dominance of the hand) [140,142,144,145].

Regarding surface characteristics, studies have shown that rough or porous substrates can retain a greater number of epithelial cells, while smooth or non-porous surfaces inhibit cell adhesion and retention [146]. For example, clothing and fabrics tend to retain more DNA than plastic or glass surfaces [147]. Conversely, metal surfaces are particularly challenging for DNA recovery due to interactions between nucleic acids and metal ions [148].

In most forensic laboratories, the analysis of contact DNA still relies on standard procedures for low-level samples, including DNA extraction and quantification before amplification, often due to internal or accreditation requirements (e.g., FBI Quality Assurance Standard 9.4) [149]. However, DNA extraction and quantification may lead to sample loss (approximately 20–90%) and increase the risk of introducing exogenous DNA [150]. Moreover, conventional PCR amplification of touch DNA often results in low-quality profiles; in approximately 69% of analyzed samples, no usable results were obtained [151]. The presence of cell-free DNA in the supernatant has been observed in 90% of biological samples, calling into question the necessity of the extraction step for touch DNA [152].

To address this, direct PCR amplification has been proposed as an alternative to avoid DNA loss during processing. This technique allows for DNA amplification from approximately 17 cells, excluding cell-free DNA [153]. Direct PCR has been used for reference samples in forensic settings since the mid-2000s, when STR amplification kits with specialized buffers to prevent PCR inhibition became available [142]. The main commercial providers offer direct PCR-compatible STR kits, such as: Thermo Fisher Scientific (Waltham, MA, USA): AmpFlSTR^®^ Identifiler^®^ Direct, NGM SElect™ Express, GlobalFiler^®^ Express, and Yfiler^®^ Direct; Promega (Madison, WI, USA): PowerPlex^®^ 18D, with all current product lines supporting direct PCR protocols, and; QIAGEN^®^ (Valencia, CA, USA): Investigator^®^ IDplex GO! and Investigator^®^ 24plex GO! kits [141].

Direct PCR offers several benefits, including increased sensitivity for trace samples, reduced costs, minimized stochastic effects, and no increase in amplification artifacts when standard cycle numbers are used [141,142,148,153]. Additionally, it has proven applicable to a variety of forensic samples, such as fabrics, hairs, and nails, processed using swabs, tape lifts, or direct application of the sample as a PCR template [151]. The success rate of profiles generated from contact DNA ranges from 33% to 100%, depending on the type of sample and amplification kit used [141]. However, when selecting a sample processing method, it is crucial to consider the potential presence of PCR inhibitors (e.g., indigo carmine dye) [154], as well as the possibility of complex DNA mixtures [142].

Furthermore, transfer DNA evidence is affected by stochastic fluctuations commonly seen in low-template samples, including high stutter, allele imbalance, and allele dropout, which complicate forensic interpretation [142]. The presence of non-self DNA on an individual’s body or clothing has also been documented. Moreover, DNA transfer at crime scenes may not always result from direct contact; secondary transfer can occur through intermediaries such as objects or other individuals. This indirect transfer can occur in two main ways: from skin to object to another person or from person to object to skin [155].

One of the main concerns in the forensic analysis of low-template DNA samples, such as those obtained from touch DNA, is the risk of contamination with exogenous human DNA, which can compromise the interpretation of results and potentially lead to spurious profiles. To address this issue, the implementation of quality standards and contamination prevention practices is critical. The ISO 18385:2016 standard (Minimizing the risk of human DNA contamination in products used to collect, store and analyze biological material for forensic purposes) was specifically developed to ensure that consumables used in forensic genetics, such as swabs, tubes, pipette tips, and reagents, are free from detectable human DNA, thereby reducing the risk of contamination from the manufacturing process [156]. This standard establishes strict production criteria, environmental control measures, personnel monitoring, and product validation requirements, including testing to confirm the absence of amplifiable DNA and exclusion databases for manufacturing personnel [157].

The use of ISO 18385-certified products has been widely recommended by organizations such as the European Network of Forensic Science Institutes (ENFSI), which promotes the adoption of this standard as part of quality assurance programs in forensic DNA laboratories [158]. The adoption of ISO 18385, alongside other quality management systems such as ISO/IEC 17025, has significantly contributed to strengthening the reliability of forensic DNA analyses and reducing contamination-related incidents, particularly in cases involving low-template DNA samples [159].

In summary, while touch DNA can yield satisfactory forensic results, the likelihood of successful profiling is significantly influenced by various factors, including donor characteristics, surface type, and laboratory methods. Therefore, further research is needed to optimize sampling strategies and analytical techniques to ensure high-quality genetic profiles and increase the evidentiary value of touch DNA in forensic investigations.

### 3.7. Forensic Genetic Genealogy

Forensic Genetic Genealogy (FGG) is an emerging practice that integrates DNA analysis, genealogical databases, and statistical methods to identify suspects or victims in criminal cases by tracing their relatives. FGG also facilitates kinship determination in adoption cases and family reunification, even between previously unknown individuals [160,161].

**Figure 6:**
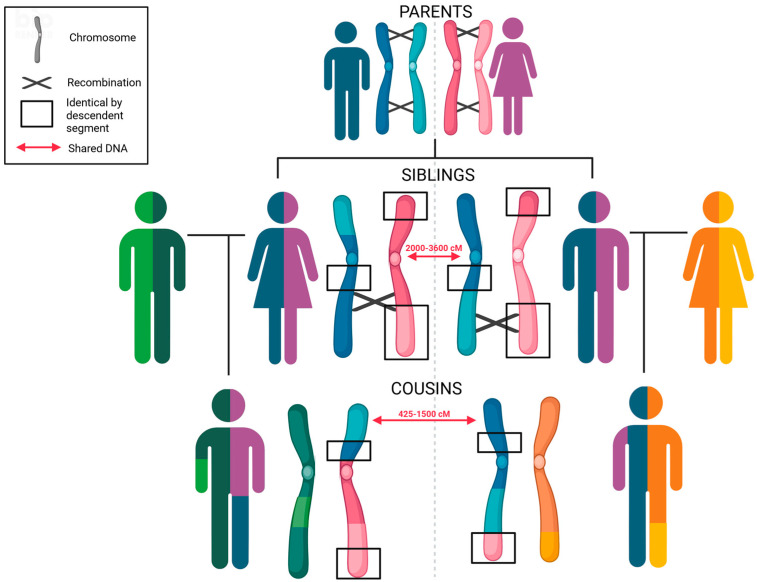
The size of IBD chromosomal segments decreases due to recombination across generations. The length of IBD segments is measured in centiMorgans (cM).

In FGG, unlike genetic genealogy, the initial sample originates from forensic evidence or biological material from a crime scene. The contracted company analyzes 600,000 to 1 million SNPs to identify identical by descent (IBD) chromosomal segments between individuals [162,163]. Due to recombination and random segregation during gamete formation, the number and length of IBD segments decrease as the generational distance increases [162,164], whereas the total sum of IBD segments increases with closer relationships (Figure 6). In addition, IBD segments can be associated with specific biogeographic ancestries, providing valuable information for genotyping service customers [162,165].

Subsequently, genomic data are uploaded to GEDmatch, a public platform where individuals can share their results, regardless of the company that conducted the genomic analysis. This platform facilitates genealogical investigations through automated mapping algorithms, enhancing the utility of genetic data [164,166]. Currently, GEDmatch hosts approximately 2 million profiles and is used by over 400 million users [163,167]. GEDmatch facilitates genealogical research by identifying Most Recent Common Ancestors (MRCA) among matching profiles, enabling the construction of family trees and networks to potentially identify a forensic sample or narrow the search to close relatives [161]. This kinship information is then supplemented with traditional genealogy, involving searching of national identity databases, vital records from government institutions and churches, and other historical sources. Nowadays, social media platforms and online genealogical resources like FamilySearch are also widely used for this purpose [161,164]. Once potential candidates are identified, kinship confirmation is often required through STR analysis, including autosomal STRs, Y-STRs, X-STRs, and even mtDNA [167]. This phase of verification may also involve searching restricted forensic databases, such as CODIS, to validate some genetic relationships [161].

In 2018, law enforcement used GEDmatch to locate and arrest the serial killer Joseph DeAngelo, also known as the Golden State Killer, whose crimes remained unsolved for over 40 years. This was the first reported use of genealogical databases in criminal investigations [167,168]. This event marked a turning point in the availability and application of genetic genealogy data for solving criminal cases and missing persons investigations. This case highlighted one of the greatest strengths provided by the FGG: the ability to rapidly solve cold cases, even those that had remained open for decades [160].

Currently, GEDmatch can be used by law enforcement agencies, notifying users that their data may be accessed for (1) violent crime investigations and (2) human remains identification, with the no-participation option in violent crime investigations [164,166]. This led to the creation of GEDmatchPro, a platform designed exclusively for forensic applications, integrating services that support FGG, such as FamilyTreeDNA and DNASolve [161,168]. Verogen, the current owner of GEDmatchPro, offers the ForenSeq Kintelligence kit, which analyzes 10,230 SNPs, including 9867 applicable to FGG, 56 ancestry-informative SNPs, 24 phenotype-informative SNPs, 94 identity-informative SNPs, 106 X-chromosome SNPs, and 85 Y-chromosome SNPs. This dataset allows for the calculation of a kinship coefficient, analogous to the total sum of IBD segments [163]. Additionally, forensic panels such as FORENSIC Capture Enrichment (FORCE) have been developed specifically for FGG applications, enabling genomic data extraction from forensic samples through the analysis of 5422 SNPs [169].

In 2017, the Brazilian government approved an initiative to collect genomic profiles from all individuals prosecuted for a crime, resulting in a database containing over 207,000 profiles by 2023. This measure contributed to a reduction in crime rates, served as a countermeasure to the country’s public security crisis, and demonstrated the potential of FGG. However, despite its effectiveness in solving cold cases, DNA database queries only generate investigative leads, requiring further investigation to confirm the individual’s identity [170].

FGG lacks a robust legal framework, hindering its systematic use even in developed nations. This presents an opportunity for countries beginning to explore its potential, but legislative formalization is complicated by privacy concerns surrounding genomic and documentary data, potentially exposing entire families. Thus, including personal genomic information in FGG databases will likely become a complex ethical decision [166,168].

The advancement of FGG requires standardization of analytical methodologies, improved data interpretation, and access to comprehensive and reliable genomic databases. For instance, the frequent contamination and degradation of forensic samples often yield insufficient genomic data for accurate distant relationship determination [162]. This limitation has hindered the widespread adoption of FGG by law enforcement agencies. Moreover, FGG’s global applicability is restricted by the underrepresentation of diverse worldwide populations in existing databases and SNP panels. Addressing this requires developing more inclusive next-generation sequencing panels and advancing computational models and artificial intelligence (AI) for haplotype reconstruction to ensure equitable and efficient application of FGG in forensic science [163].

### 3.8. Rapid DNA Profiling

**Figure 7:**
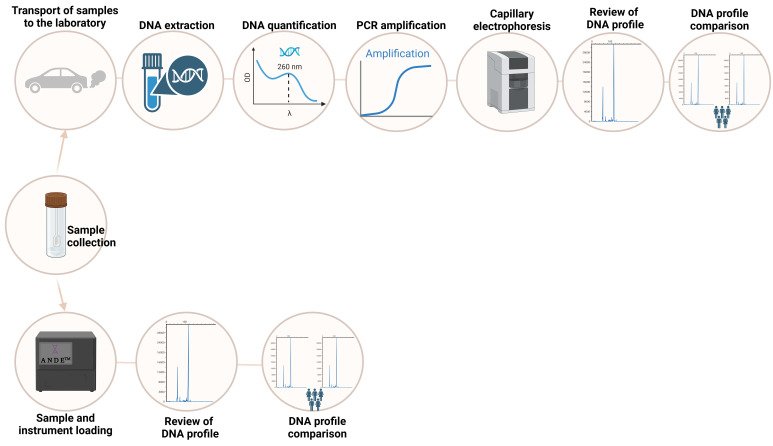
Flowchart describing the necessary phases for processing a sample collected for forensic purposes, comparing the traditional analysis method (**above**) with the rapid DNA profiling method (**below**).

Rapid DNA profiling is the application of advanced techniques that enable the generation of genetic profiles in a significantly reduced time. It emerges as a tool to optimize the genetic identification process by decreasing the time required compared to traditional STR analysis methods, where the conventional procedure involves multiple stages, such as DNA extraction, quantification of the extracted genetic material, amplification via PCR, fragment separation by capillary electrophoresis, interpretation of the obtained profiles, and comparison with forensic databases. In contrast, rapid DNA profiling employs automated and portable technology based on an all-in-one consumable chip system, allowing for direct analysis of forensic samples such as buccal swabs or bloodstains [171] (Figure 7).

With this method, a genetic profile can be obtained in less than two hours, depending on the sample quality and the equipment used. This facilitates its application in criminal investigations, victim identification in natural disasters, military operations, kinship testing in forensic contexts, and identity verification in border control. As an automated system, the user, who does not require specialized scientific training, only needs to load the sample into the processing chamber and activate the device, which performs the analysis autonomously [172].

Among the most representative rapid DNA profiling systems compatible with the CODIS system, the following stand out: IntegenX RapidHIT 200, a rapid DNA profiling instrument acquired by Thermo Fisher Scientific in 2018. It can process up to eight samples simultaneously, generating genetic profiles in under two hours. Its compatibility with CODIS and the use of disposable cartridges allow for intuitive operation, with an accessible menu that reduces the need for specialized training. Its design optimizes the generation of matching profiles, especially from buccal samples, through the “swab in, profile out” process [173], and ANDE™ 6C Rapid DNA Analysis™ by ANDE Corporation is a system designed for forensic applications and can process up to five samples simultaneously, ensuring the absence of cross-contamination and enabling the analysis of 27 STR loci, which enhances the accuracy of genetic profile identification and mixture detection [174].

The implementation of rapid DNA profiling systems in forensic settings presents both ethical and legal challenges and limitations. It is crucial to consider the specific legislation and regulations of each country regarding its application, as well as principles of privacy and personal data protection. Additionally, assessing the admissibility of genetic profiles generated by these technologies in judicial proceedings is essential, ensuring compliance with recognized forensic standards. Furthermore, potential sources of error in identification must be analyzed, including sensitivity to degraded samples, handling of DNA mixtures, and interpretation of results in complex forensic contexts [175].

### 3.9. Non-Human DNA Typing

Investigation of non-human DNA or wildlife is a branch of forensic genetics that studies the animal and plant taxonomy present in evidence, which helps to identify crimes against wildlife, especially in countries rich in biodiversity [176]. Specifically, according to the Society for Wildlife Forensic Science (SWFS), wildlife forensic science is *the application of a range of scientific disciplines to legal cases involving non-human biological evidence*. This discipline has developed alongside human forensic genetics, utilizing similar techniques and statistical methods [177].

Wildlife forensics can be divided into four main objectives: species identification (including sex determination), geographic origin, biological relationship and individual identification [176,178,179]. For each of these objectives, various techniques are applied, which we will briefly discuss in this section. Species identification from an evidence sample is the most common application in wildlife DNA forensics [176]. For this purpose, DNA regions with variation between species but conserved within the same species are analyzed. In animals, one of the most commonly used regions is the cytochrome b gene and subunit I of cytochrome oxidase (mitochondrial genome) [178,179], while for plants, the chloroplast DNA sequence is analyzed. The most commonly used reference databases for comparative species identification searches [180] are the NCBI/EMBL/DDBJ database collaboration (www.insdc.org) and BOLD, part of the Consortium for the Barcoding of Life (CBOL, www.barcodinglife.com) [176]. Currently, MPS platforms have been validated for the identification of non-human DNA, especially for species identification [181,182].

Identifying the geographic origin of a species is based, as in humans, on the use of genetic markers that allow populations to be differentiated and/or that are related to a geographic origin. For this purpose, the existence of population databases is always necessary [183]. It has been studied that mtDNA can be used to identify populations and geographic origins [176]. Some genetic markers (STRs and SNPs of the nuclear genome) have also been used, but this requires obtaining allele frequencies to characterize their genetic structure [183].

As highlighted in the section on wildlife forensic genetics, the use of DNA-based reference data is crucial for species identification, sex determination, and geographic origin analysis in wildlife crime investigations. Although the existence of individual profile databases for some endangered species like big cats, rhinos, or elephants is not directly discussed in this review, the text acknowledges the need for comprehensive reference datasets. Currently, tools such as mitochondrial DNA analysis, STRs, SNPs, and resources like the Feather Atlas represent important databasing efforts that contribute to the identification of trafficked wildlife specimens. In addition, in some cases, such as poaching, animal theft, or even animal attacks on humans, it is necessary to confirm that an animal part (bone, skin, tusk) belongs to a specific individual. For this purpose, DNA markers that vary between species (STRs, SNPs and INDELs) are used [184,185,186]. Individual DNA profiles can also be used to prevent trade in protected species through DNA registries, where specimens can be individually identified by DNA profiling [177]. Another important application of wildlife forensic genetics involves distinguishing captive-bred individuals from those captured in the wild, which helps prevent the illegal trade of rare species falsely claimed as captive-bred [187]. The development and validation of such databases remain a significant challenge and an area where future work is needed.

One of the main challenges in wildlife DNA forensics is the availability of validated reference genetic data, both for DNA reference sequence databases and individual profiles [188]. This is because most databases are generated internally, and some laboratories lack the necessary resources to create their databases. Therefore, to address the continued use of databases like GenBank in forensic cases, the Standard for the Selection and Evaluation of GenBank Results for Wildlife Taxonomic Assignment (ANSI/ASB Standard 180, 2024) was created [189].

Finally, another challenge facing the wildlife research field is the lack of funding, as not all countries have the resources to combat wildlife crime without foreign funding [176]. In addition, time and expertise are other limitations, as validating new technologies for forensic use can take months or years [190].

## 4. Future Perspectives

Standard PCR-CE technology based on the analysis of genetic systems that include at least the 20 CODIS STRs allows obtaining values with sufficient statistical power to resolve most human identification cases [191]. However, when it is impossible to obtain complete or conclusive results through conventional technology, MPS represents a promising alternative. For example, MPS has proven to be useful for the differentiation of monozygotic twins (through the analysis of SNPs, CNVs, and epigenetic markers) [192] and cases of paternity with the presence of mutations [193]. Unfortunately, these technologies have not been routinely adopted in forensic laboratories yet, both because of the higher cost and lack of strategies and methodologies to deal with large amounts of information generated by genomic platforms. In this regard, the recommendations of the DNA Commission of the International Society for Forensic Genetics (ISFG) for the publication of sequence-based STR findings were recently published [194], which aim to regulate and standardize the way of reporting STR data based on MPS technologies. This work could accelerate the implementation of this technology in the forensic area, allowing improved estimates of biological kinship relationships or identification of biological samples found at crime scenes.

Gradual implementation of MPS for the resolution of forensic cases could be favored by the steady development of commercially available forensic genomics-based kits, such as the ForenSeq DNA Signature Prep kit [195], Precision ID GlobalFiler™ NGS STR Panel [196], Promega PowerSeq™ Auto/Y system [197], and PowerSeq™ 46GY System [198], among others.

On the other hand, other human identification tools have also been enhanced through MPS, such as mtDNA, from which it is now possible to sequence the complete mitochondrial genome, which has been shown to generate greater discriminatory power concerning the analysis of hypervariable regions of the control region [199], even in degraded samples or with a low amount of initial DNA [200]. Although it is still of great importance to build databases of mitochondrial genomes that make it possible to perform human identification calculations based on these genomic tools. Furthermore, genomic tools have also been designed for the inference of phenotypes or externally visible characteristics (EVCs), as is the case of the ForenSeq IMAGE system [201], which allows the prediction of eye, skin, and hair color (together with the analysis of Y-SNPs and ancestry-informative SNPs). However, studies to predict phenotypes in different populations around the world are very important, given the large variability in accuracy that has been observed in prediction results between different populations around the world [149,150,151,152,153,154]. In addition, although strategies to predict other characteristics, such as the habit of consuming drugs like tobacco or alcohol have already been published [131], it is possible that in the coming years, these strategies will be improved or even commercial kits will be developed, which would facilitate their implementation in legal cases.

There have also been pilot studies that could be very useful in the future, such as the “Sexome”, which involves the analysis through MPS of the bacterial microbiome obtained from the post-coital genital area to identify possible aggressions or sexual contacts in legal cases [132]. Although this technique is possibly limited in that it loses effectiveness if the area is cleaned after contact, it can be a very good alternative in cases of rape investigations when the aggressor is azoospermic or used a condom. Another technique that is gaining great relevance and will possibly be very useful in the future in the forensic area is the application of CRISPR-Cas, which consists of the use of guide RNA (gRNA) complementary to the target DNA sequence, using proteins from the immune system of bacteria, the Clustered Regularly Interspaced Short Palindromic Repeats (CRISPR) along with CRISPR-associated (Cas) proteins to precisely target and modify specific sequences of DNA [202]. As observed in several other scientific areas, CRISPR-Cas technology is emerging as a novel tool with potential applications for human identification purposes. DNA profiling and identification is likely to be one of the most significant applications of CRISPR-Cas technology in forensic sciences, perhaps reducing steps and costs [203] by providing higher sensitivity in analyzing DNA evidence than traditional methods’ sensitivity limits [204]; moreover, it also has the potential to be used on-site using portable DNA biosensor devices. This allows for overcoming degraded or low-input DNA limitations, thus enhancing forensic analysis [204].

Finally, an emerging area that is evolving rapidly is the implementation of artificial intelligence (AI) and machine learning (ML) in forensic genetics, where strategies for correct allele assignment and stutter detection stand out, more efficient detection of the number of co-contributors in a biological mixture, improved prediction of phenotypic characteristics and biogeographic ancestry, more accurate analysis of the microbiome to infer the post-mortem interval in cadavers, as well as prediction of Y-chromosome haplogroups from Y-STR haplotypes [205,206]. Both AI and ML technologies enable forensic scientists to analyze DNA evidence more rapidly and with fewer errors, reducing casework and accelerating justice [207].

## 5. Conclusions

Forensic genetics has undergone remarkable evolution, transitioning from basic applications in individual identification to a cornerstone of complex, multidisciplinary investigations. Early methods, such as blood group and protein analysis, laid the foundation for the field. However, the advent of DNA technologies has revolutionized forensic science, offering unprecedented precision and reliability. Modern tools, MPS, and the analysis of genetic markers like STRs and SNPs and forensic epigenetics have transformed the ability to resolve complex cases, identify victims in mass disasters, and address a myriad of ethical, legal, and technical challenges. Furthermore, the integration of international databases has significantly enhanced global collaboration and data sharing, strengthening the reach and impact of forensic genetics.

Looking ahead, the future of forensic genetics lies in a more interdisciplinary and personalized approach. Emerging technologies, such as artificial intelligence and machine learning, promise to refine genomic data analysis, while the discovery of novel genetic markers and the development of advanced commercial kits will further expand the field’s applications in human identification. Nonetheless, this progress must be tempered by efforts to address critical challenges, including the protection of genetic data privacy, the standardization of methodologies, the need for more diverse population databases, and ensuring equitable access to these technologies across diverse regions and populations.

In summary, forensic genetics will continue to be an indispensable tool in the pursuit of justice and truth. Its ongoing evolution will depend on collaboration among scientists, policymakers, and legal professionals, ensuring that technological advancements are applied responsibly, ethically, and for the benefit of society.

## Figures and Tables

**Table 1:** Main commercial autosomal STR kits that have been validated for forensic application.

Name	Source	STRs and Other Markers Included	Reference
AmpFlSTR^®^ COfiler™	Applied Biosystems	D3S1358, D16S539, Amelogenin, TH01, TPOX, CSF1PO, D7S820	[59]
AmpFlSTR^®^ Profiler™	Applied Biosystems	D3S1358, vWA, FGA, Amelogenin, TH01, TPOX, CSF1PO, D5S818, D13S317, D7S820	[59]
AmpFISTR^®^ Identifiler™	Applied Biosystems	D8S1179, D21S11, D7S820, CSF1PO, D3S1358, TH01, D13S317, D16S539, D2S1338, D19S433, vWA, TPOX, D18S51, Amelogenin, D5S818, FGA	[60]
GlobalFiler™	Applied Biosystems	D13S317, D7S820, D5S818, CSF1PO, D1S1656, D12S391, D2S441, D10S1248, D18S51, FGA, D21S11, D8S1179, vWA, D16S539, TH01, D3S1358, AMEL, D2S1338, D19S433, DYS391, TPOX, D22S1045, SE33 and a Y-specific insertion/deletion locus (Y-indel).	[61]
Investigator IDplex Plus Kit	QIAGEN	CSF1PO, FGA, TH01, TPOX, VWA, D3S1358, D5S818, D7S820, D8S1179, D13S317, D16S539, D18S51, and D21S1, D2S1338, D19S433 and Amelogenin.	[62]
PowerPlex^®^ 1.2	Promega	D16S539, D7S820, D13S317, D5S818, CSF1PO, TPOX, TH01, vWA.	[63]
PowerPlex^®^ 16 Monoplex System	Promega	D3S1358, TH01, D21S11, D18S51, Penta E, D5S818, D13S317, D7S820, D16S539, CSF1PO, Penta D, Amelogenin, vWA, D8S1179, TPOX, FGA.	[64]
The PowerPlex(^®^) 21 System	Promega	D1S1656, D2S1338, D3S1358, D5S818, D6S1043, D7S820, D8S1179, D12S391, D13S317, D16S539, D18S51, D19S433, D21S11, Amelogenin, CSF1PO, FGA, Penta D, Penta E, TH01, TPOX, and vWA.	[65]
PowerPlex^®^ fusion 5C	Promega	Amelogenin, D3S1358, D1S1656, D2S441, D10S1248, D13S317, Penta E, D16S539, D18S51, D2S1338, CSF1PO, Penta D, TH01, vWA, D21S11, D7S820, D5S818, TPOX, DYS391, D8S1179, D12S391, D19S433, FGA, and D22S1045.	[66]
PowerPlex^®^ fusion 6C	Promega	CSF1PO, FGA, TH01, TPOX, vWA, D1S1656, D2S1338, D2S441, D3S1358, D5S818, D7S820, D8S1179, D10S1248, D12S391, D13S317, D16S539, D18S51, D19S433, D21S11 and D22S1045, Amelogenin and DYS391 for gender determination, Penta D, Penta E and SE33.	[67]
VersaPlex™ 27PY	Applied Biosystems	CSF1PO, FGA, TH01, TPOX, vWA, D1S1656, D2S1338, D2S441, D3S1358, D5S818, D7S820, D8S1179, D10S1248, D12S391, D13S317, D16S539, D18S51, D19S433, D21S11, D22S1045, Amelogenin, DYS391, Penta D, Penta E, D6S1043, DYS570 and DYS576.	[68]
Investigator 24plex QS	QIAGEN	D1S1656, D2S441, D2S1338, D3S1358, D5S818, D7S820, D8S1179, D10S1248, D12S391, D13S317, D16S539, D18S51, D19S433, D21S11, D22S1045, CSF1PO, FGA, TH01, TPOX, vWA, SE33, DYS391, Amelogenin and the Quality Sensor.	[69]

## References

[1] Shen M., Vieira D.N. (2016). Forensic Science: Defending Justice. Forensic Sci. Res..

[2] de Knijff P. (2019). From next generation sequencing to now generation sequencing in forensics. Forensic Sci. Int. Genet..

[3] Butler J.M., Buel E., Crivellente F., McCord B.R. (2004). Forensic DNA typing by capillary electrophoresis using the ABI Prism 310 and 3100 genetic analyzers for STR analysis. Electrophoresis.

[4] MacLean C.E., Lamparello A. (2014). Forensic DNA phenotyping in criminal investigations and criminal courts: Assessing and mitigating the dilemmas inherent in the science. Recent Adv. DNA Gene Seq..

[5] Kayser M., Siegel J.A., Saukko P.J. (2013). Forensic DNA Phenotyping: DNA testing for externally visible characteristics. Encyclopedia of Forensic Sciences.

[6] Landsteiner K. (1900). Zur Kenntnis der Anti-Fermentatiren, Lytrschen and Agglutinierenden Wirkungen des Bluteerums und der Lymphe. Zentralbi Bakteriol.

[7] Bugert P., Klüter H. (2012). 100 Years after von Dungern & Hirschfeld: Kinship Investigation from Blood Groups to SNPs. Transfus. Med. Hemother. Off. Organ Dtsch. Ges. Transfusionsmedizin Immunhamatol..

[8] Landsteiner K., Levine P. (1928). On individual differences in human blood. J. Exp. Med..

[9] Landsteiner K., Levine P. (1928). On the inheritance of agglutinogens of human blood demonstrable by immune agglutinins. J. Exp. Med..

[10] Landsteiner K., Wiener A.S. (1941). Studies on an agglutinogen (rh) in human blood reacting with anti-rhesus sera and with human isoantibodies. J. Exp. Med..

[11] Sussman L.N. (1954). Blood grouping tests in disputed paternity proceedings; studies with ABO, MN, and Rh-Hr factors. J. Am. Med. Assoc..

[12] Sussman L.N. (1963). Blood grouping tests. A review of 1000 cases of disputed paternity. Am. J. Clin. Pathol..

[13] Wiener A.S., Sonn E.B., Belkin R.B. (1944). Heredity of the rh blood types. J. Exp. Med..

[14] Landsteiner K. (1934). Forensic application of serologic individuality tests. J. Am. Med. Assoc..

[15] Boyd W.C. (1949). Use of blood groups in cases of disputed paternity. N. Engl. J. Med..

[16] Cerda-Flores R.M., Barton S.A., Marty-Gonzalez L.F., Rivas F., Chakraborty R. (1999). Estimation of nonpaternity in the Mexican population of Nuevo Leon: A validation study with blood group markers. Am. J. Phys. Anthropol..

[17] Harley D., Lynch G.R. (1937). Blood Tests for Paternity. Br. Med. J..

[18] Dausset J. (1958). Iso-leuko-antibodies. Acta Haematol..

[19] Terasaki P.I. (1978). HLA in paternity testing. West. J. Med..

[20] Lee C.L., Lebeck L.K., Wong C. (1980). Estimating paternity index from HLA-typing results. Am. J. Clin. Pathol..

[21] Singh G., Johns M.M., Paul G. (1982). Paternity testing: Analysis of six blood groups and HLA markers, with particular reference to comparison of races. Am. J. Clin. Pathol..

[22] Borowsky R. (1988). HLA and the probability of paternity. Am. J. Hum. Genet..

[23] Davey F.R., Hubbell C.A., Lauenstein K.J., Tinnesz C., Henry J.B. (1984). Analysis of paternity. The use of HLA and red cell antigens. Transfusion.

[24] Houtz T.D., Brooks M.A., Wenk R.E., Dawson R.B. (1981). Utility of HLA and six erythrocyte antigen systems in excluding paternity among 500 disputed cases. Forensic Sci. Int..

[25] Reisner E.G., MacQueen J.M. (1981). Problems arising from the use of the HLA system in paternity testing. Clin. Lab. Haematol..

[26] Butler J.M. (2005). Forensic DNA Typing: Biology, Technology, and Genetics of STR Markers.

[27] Smithies O. (1955). Zone electrophoresis in starch gels: Group variations in the serum proteins of normal human adults. Biochem. J..

[28] Dykes D.D., Polesky H.F. (1976). The usefulness of serum protein and erythrocyte enzyme polymorphisms in paternity testing. Am. J. Clin. Pathol..

[29] Spencer N., Hopkinson D.A., Harris H. (1964). Phosphoglucomutase polymorphism in man. Nature.

[30] Fuhrmann W., Lichte K.H. (1966). Human red cell acid phosphatase polymorphism. A study on gene frequency and forensic use of the system in cases of disputed paternity. Humangenetik.

[31] Parr C.W. (1966). Erythrocyte phosphogluconate dehydrogenase polymorphism. Nature.

[32] Seppälä M., Ruoslahti E., Seppälä I.J. (1967). Transferrin system in paternity cases. An immunological verification test for heterozygosity. Ann. Med. Exp. Biol. Fenn..

[33] McCombs M.L., Bowman B.H. (1969). Demonstration of inherited ceruloplasmin variants in human Serum by acrylamide electrophoresis. Tex. Rep. Biol. Med..

[34] Harris H. (1971). Protein polymorphism in man. Can. J. Genet. Cytol. J. Can. Genet. Cytol..

[35] Lefèvre H., Fiedler H., Niebuhr R. (1972). Value of the adenosine deaminase (ADA) isoenzyme system for forensic evidence: Statistical dependability testing of paternity exclusion claims. Z. Rechtsmed. J. Leg. Med..

[36] Bender K., Volkmann E. (1970). Paternity exclusion using the transferrin system. Z. Immun. Allerg. Klin. Immunol..

[37] Waldinger D., Cleve H. (1988). Two-dimensional electrophoresis of human lymphocyte proteins: Two-dimensional polymorphisms and paternity testing. Electrophoresis.

[38] Salmon D.B., Brocteur J. (1978). Probability of paternity exclusion when relatives are involved. Am. J. Hum. Genet..

[39] Saad R. (2005). Discovery, development, and current applications of DNA identity testing. Proceedings.

[40] Aronson J.D. (2005). DNA fingerprinting on trial: The dramatic early history of a new forensic technique. Endeavour.

[41] Wyman A.R., White R. (1980). A highly polymorphic locus in human DNA. Proc. Natl. Acad. Sci. USA.

[42] Jeffreys A.J., Wilson V., Thein S.L. (1985). Hypervariable ‘minisatellite’ regions in human DNA. Nature.

[43] Jeffreys A.J., Wilson V., Thein S.L. (1985). Individual-specific ‘fingerprints’ of human DNA. Nature.

[44] Lindblom B., Holmlund G., Stennek A. (1991). DNA analyses in forensic investigations. Lakartidningen.

[45] Tautz D. (1993). Notes on the definition and nomenclature of tandemly repetitive DNA sequences. EXS.

[46] Katti M.V., Ranjekar P.K., Gupta V.S. (2001). Differential distribution of simple sequence repeats in eukaryotic genome sequences. Mol. Biol. Evol..

[47] Jeffreys A.J., Brookfield J.F., Semeonoff R. (1985). Positive identification of an immigration test-case using human DNA fingerprints. Nature.

[48] Kayser M. (2015). Forensic DNA Phenotyping, Predicting human appearance from crime scene material for investigative purposes. Forensic Sci. Int. Genet..

[49] Ellegren H. (2004). Microsatellites: Simple sequences with complex evolution. Nat. Rev. Genet..

[50] Collins J.R., Stephens R.M., Gold B., Long B., Dean M., Burt S.K. (2003). An exhaustive DNA micro-satellite map of the human genome using high performance computing. Genomics.

[51] Panneerchelvam S., Norazmi M.N. (2003). Forensic DNA profiling and database. Malays. J. Med. Sci..

[52] Butler J.M., Butler J.M., San D. (2012). Chapter 5—Short Tandem Repeat (STR) Loci and Kits. Advanced Topics in Forensic DNA Typing: Methodology.

[53] Butler J.M. (2006). Genetics and genomics of core short tandem repeat loci used in human identity testing. J. Forensic Sci..

[54] Budowle B., Moretti T.R., Niezgoda S.J., Brown B.L. (1998). CODIS and PCR-based short tandem repeat loci: Law enforcement tools. Proceedings of the Second European Symposium on Human Identification.

[55] Nwawuba Stanley U., Mohammed Khadija A., Bukola A.T., Omusi Precious I., Ayevbuomwan Davidson E. (2020). Forensic DNA Profiling: Autosomal Short Tandem Repeat as a Prominent Marker in Crime Investigation. Malays. J. Med. Sci..

[56] ENFSI Standing Committee for Quality and Competence (QCC) Validation and Implementation of (New) Methods; 2006. Ref. Code: QCC-VAL-001. http://www.enfsi.eu.

[57] Scientific Working Group on DNA Analysis Methods (SWGDAM) (2012). Validation Guidelines for DNA Analysis Methods.

[58] Hares D.R. (2015). Selection and implementation of expanded CODIS core loci in the United States. Forensic Sci. Int. Genet..

[59] LaFountain M.J., Schwartz M.B., Svete P.A., Walkinshaw M.A., Buel E. (2001). TWGDAM validation of the AmpFlSTR Profiler Plus and AmpFlSTR COfiler STR multiplex systems using capillary electrophoresis. J. Forensic Sci..

[60] Collins P.J., Hennessy L.K., Leibelt C.S., Roby R.K., Reeder D.J., Foxall P.A. (2004). Developmental validation of a single-tube amplification of the 13 CODIS STR loci, D2S1338, D19S433, and amelogenin: The AmpFlSTR Identifiler PCR Amplification Kit. J. Forensic Sci..

[61] Ludeman M.J., Zhong C., Mulero J.J., Lagacé R.E., Hennessy L.K., Short M.L., Wang D.Y. (2018). Developmental validation of GlobalFiler™ PCR amplification kit: A 6-dye multiplex assay designed for amplification of casework samples. Int. J. Leg. Med..

[62] Mattayat D., Kitpipit T., Phetpeng S., Asawutmangkul W., Thanakiatkrai P. (2016). Comparative performance of AmpFLSTR^®^ Identifiler^®^ Plus PCR amplification kit and QIAGEN^®^ Investigator^®^ IDplex Plus kit. Sci. Justice J. Forensic Sci. Soc..

[63] Levedakou E.N., Freeman D.A., Budzynski M.J., Early B.E., Damaso R.C., Pollard A.M., Townley A.J., Gombos J.L., Lewis J.L., Kist F.G. (2002). Characterization and validation studies of powerplex 2.1, a nine-locus short tandem repeat (STR) multiplex system and penta D monoplex. J. Forensic Sci..

[64] Krenke B.E., Tereba A., Anderson S.J., Buel E., Culhane S., Finis C.J., Tomsey C.S., Zachetti J.M., Masibay A., Rabbach D.R. (2002). Validation of a 16-locus fluorescent multiplex system. J. Forensic Sci..

[65] Ensenberger M.G., Hill C.R., McLaren R.S., Sprecher C.J., Storts D.R. (2014). Developmental validation of the PowerPlex(^®^) 21 System. Forensic Sci. Int. Genet..

[66] Oostdik K., Lenz K., Nye J., Schelling K., Yet D., Bruski S., Strong J., Buchanan C., Sutton J., Linner J. (2014). Developmental validation of the PowerPlex^®^ Fusion System for analysis of casework and reference samples: A 24-locus multiplex for new database standards. Forensic Sci. Int. Genet..

[67] Ensenberger M.G., Lenz K.A., Matthies L.K., Hadinoto G.M., Schienman J.E., Przech A.J., Morganti M.W., Renstrom D.T., Baker V.M., Gawrys K.M. (2016). Developmental validation of the PowerPlex^®^ Fusion 6C System. Forensic Sci. Int. Genet..

[68] Shrivastava P., Dixit S., Kumawat R.K., Srivastava A. (2022). Efficiency analysis of VersaPlex™ 27PY system in Central Indian Population: First report from Indian population. Leg. Med..

[69] Kraemer M., Prochnow A., Bussmann M., Scherer M., Peist R., Steffen C. (2017). Developmental validation of QIAGEN Investigator^®^ 24plex QS Kit and Investigator^®^ 24plex GO! Kit: Two 6-dye multiplex assays for the extended CODIS core loci. Forensic Sci. Int. Genet..

[70] Martin P.D., Schmitter H., Schneider P.M. (2001). A brief history of the formation of DNA databases in forensic science within Europe. Forensic Sci. Int..

[71] Ruitberg C.M., Reeder D.J., Butler J.M. (2001). STRBase: A short tandem repeat DNA database for the human identity testing community. Nucleic Acids Res..

[72] Roewer L., Krawczak M., Willuweit S., Nagy M., Alves C., Amorim A., Anslinger K., Augustin C., Betz A., Bosch E. (2001). Online reference database of European Y-chromosomal short tandem repeat (STR) haplotypes. Forensic Sci. Int..

[73] Bodner M., Bastisch I., Butler J.M., Fimmers R., Gill P., Gusmão L., Morling N., Phillips C., Prinz M., Schneider P.M. (2016). Recommendations of the DNA Commission of the International Society for Forensic Genetics (ISFG) on quality control of autosomal Short Tandem Repeat allele frequency databasing (STRidER). Forensic Sci. Int. Genet..

[74] Sobrino B., Carracedo A. (2005). SNP typing in forensic genetics: A review. Methods Mol. Biol..

[75] Butler J.M., Butler J., Walthan M.A. (2012). Single Nucleotide polymorphisms and Applications. Advanced Topics in Forensic DNA Typing: Methodology.

[76] Phillips C. (2012). Application of Autosomal SNPs and Indels in Forensic Analysis. Forensic Sci. Rev..

[77] Budowle B., van Daal A. (2008). Forensically relevant SNP classes. Biotechniques.

[78] Malkki M., Petersdorf E.W. (2012). Genotyping of single nucleotide polymorphisms by 5′ nuclease allelic discrimination. Methods Mol. Biol..

[79] Mehta B., Daniel R., Phillips C., McNevin D. (2017). Forensically relevant SNaPshot(^®^) assays for human DNA SNP analysis: A review. Int. J. Leg. Med..

[80] Seo S.B., King J.L., Warshauer D.H., Davis C.P., Ge J., Budowle B. (2013). Single nucleotide polymorphism typing with massively parallel sequencing for human identification. Int. J. Leg. Med..

[81] Alonso A., Barrio P.A., Müller P., Köcher S., Berger B., Martin P., Bodner M., Willuweit S., Parson W., Roewer L. (2018). Current state-of-art of STR sequencing in forensic genetics. Electrophoresis.

[82] Zidkova A., Horinek A., Kebrdlova V., Korabecna M. (2013). Application of the new insertion-deletion polymorphism kit for forensic identification and parentage testing on the Czech population. Int. J. Leg. Med..

[83] Holt C.L., Stephens K.M., Walichiewicz P., Fleming K.D., Forouzmand E., Wu S.F. (2021). Human Mitochondrial Control Region and mtGenome: Design and Forensic Validation of NGS Multiplexes, Sequencing and Analytical Software. Genes.

[84] Venables S.J., Mehta B., Daniel R., Walsh S.J., van Oorschot R.A., McNevin D. (2014). Assessment of high resolution melting analysis as a potential SNP genotyping technique in forensic casework. Electrophoresis.

[85] Palencia-Madrid L., Xavier C., de la Puente M., Hohoff C., Phillips C., Kayser M., Parson W. (2020). Evaluation of the VISAGE Basic Tool for Appearance and Ancestry Prediction Using PowerSeq Chemistry on the MiSeq FGx System. Genes.

[86] Breslin K., Wills B., Ralf A., Ventayol Garcia M., Kukla-Bartoszek M., Pospiech E., Freire-Aradas A., Xavier C., Ingold S., de La Puente M. (2019). HIrisPlex-S system for eye, hair, and skin color prediction from DNA: Massively parallel sequencing solutions for two common forensically used platforms. Forensic Sci. Int. Genet..

[87] Kiesler K.M., Borsuk L.A., Steffen C.R., Vallone P.M., Gettings K.B. (2023). US Population Data for 94 Identity-Informative SNP Loci. Genes.

[88] Fondevila M., Pereira R., Gusmão L., Phillips C., Lareu M.V., Carracedo A. (2011). Forensic performance of insertion-deletion marker systems. Forensic Sci. Int. Genet. Suppl. Ser..

[89] Manta F., Caiafa A., Pereira R., Silva D., Amorim A., Carvalho E.F., Gusmão L. (2012). Indel markers: Genetic diversity of 38 polymorphisms in Brazilian populations and application in a paternity investigation with post mortem material. Forensic Sci. Int. Genet..

[90] LaRue B.L., Ge J., King J.L., Budowle B. (2012). A validation study of the Qiagen Investigator DIPplex^®^ kit; an INDEL-based assay for human identification. Int. J. Leg. Med..

[91] Casals F., Rasal R., Anglada R., Tormo M., Bonet N., Rivas N., Vásquez P., Calafell F. (2022). A forensic population database in El Salvador: 58 STRs and 94 SNPs. Forensic Sci. Int. Genet..

[92] Aguilar-Velázquez J.A., Duran-Salazar M., Córdoba-Mercado M.F., Coronado-Avila C.E., Salas-Salas O., Martinez-Cortés G., Casals F., Calafell F., Ramos-González B., Rangel-Villalobos H. (2022). Characterization of 58 STRs and 94 SNPs with the ForenSeq™ DNA signature prep kit in Mexican-Mestizos from the Monterrey city (Northeast, Mexico). Mol. Biol. Rep..

[93] Guevara E.K., Palo J.U., King J.L., Buś M.M., Guillén S., Budowle B., Sajantila A. (2021). Autosomal STR and SNP characterization of populations from the Northeastern Peruvian Andes with the ForenSeqTM; DNA Signature Prep Kit. Forensic Sci. Int. Genet..

[94] Peng D., Zhang Y., Ren H., Li H., Li R., Shen X., Wang N., Huang E., Wu R., Sun H. (2020). Identification of sequence polymorphisms at 58 STRs and 94 iiSNPs in a Tibetan population using massively parallel sequencing. Sci. Rep..

[95] Delest A., Godfrin D., Chantrel Y., Ulus A., Vannier J., Faivre M., Hollard C., Laurent F.-X. (2020). Sequenced-based French population data from 169 unrelated individuals with Verogen’s ForenSeq DNA signature prep kit. Forensic Sci. Int. Genet..

[96] Hussing C., Bytyci R., Huber C., Morling N., Børsting C. (2019). The Danish STR sequence database: Duplicate typing of 363 Danes with the ForenSeq™ DNA Signature Prep Kit. Int. J. Leg. Med..

[97] Butler J.M., Butler J.M. (2012). Relationship Testing: Kinship Statistics. Advanced Topics in Forensic DNA Typing: Interpretation.

[98] Wein S., Scott M.S. (2024). Forensic DNA analysis and statistics. Forensic DNA Applications: An Interdisciplinary Perspective.

[99] Shrivastava P., Trivedi V.B. (2023). Calculation of paternity index in paternity dispute and identification cases. Principles and Practices of DNA Analysis: A Laboratory Manual for Forensic DNA Typing.

[100] Butler J.M., Butler J.M. (2015). DNA profile frequency estimates and match probabilities. Advanced Topics in Forensic DNA Typing: Interpretation.

[101] Prinz M., Carracedo A., Mayr W.R., Morling N., Parsons T.J., Sajantila A., Scheithauer R., Schmitter H., Schneider P.M. (2007). DNA Commission of the International Society for Forensic Genetics (ISFG): Recommendations regarding the role of forensic genetics for disaster victim identification (DVI). Forensic Sci. Int. Genet..

[102] Gusmão L., Butler J.M., Carracedo A., Gill P., Kayser M., Mayr W.R., Morling N., Prinz M., Roewer L., Tyler-Smith C. (2006). DNA Commission of the International Society of Forensic Genetics (ISFG): An update of the recommendations on the use of Y-STRs in forensic analysis. Forensic Sci. Int..

[103] Tillmar A.O., Kling D., Butler J.M., Parson W., Prinz M., Schneider P.M., Egeland T., Gusmão L. (2017). DNA Commission of the International Society for Forensic Genetics (ISFG): Guidelines on the use of X-STRs in kinship analysis. Forensic Sci. Int. Genet..

[104] Gjertson D.W., Brenner C.H., Baur M.P., Carracedo A., Guidet F., Luque J.A., Lessig R., Mayr W.R., Pascali V.L., Prinz M. (2007). ISFG: Recommendations on biostatistics in paternity testing. Forensic Sci. Int. Genet..

[105] Yan C., Duanmu X., Zeng L., Liu B., Song Z. (2019). Mitochondrial DNA: Distribution, Mutations, and Elimination. Cells.

[106] Budowle B., Allard M.W., Wilson M.R., Chakraborty R. (2003). Forensics and mitochondrial DNA: Applications, debates, and foundations. Annu. Rev. Genom. Hum. Genet..

[107] Amorim A., Fernandes T., Taveira N. (2019). Mitochondrial DNA in human identification: A review. PeerJ.

[108] Ohuchi T., Guan X., Funayama M. (2021). Evaluation of the Utility of Mitochondrial DNA Testing in Personal Identification Work in the Great East Japan Earthquake of 2011. Tohoku J. Exp. Med..

[109] Parson W., Bandelt H.J. (2007). Extended guidelines for mtDNA typing of population data in forensic science. Forensic Sci. Int. Genet..

[110] Bär W., Brinkmann B., Budowle B., Carracedo A., Gill P., Holland M., Lincoln P.J., Mayr W., Morling N., Olaisen B. (2000). Guidelines for mitochondrial DNA typing. DNA Commission of the International Society for Forensic Genetics. Vox Sang..

[111] SWGDAM (2019). Interpretation Guidelines for Mitochondrial DNA Analysis by Forensic DNA Testing Laboratories.

[112] Parson W., Gusmão L., Hares D.R., Irwin J.A., Mayr W.R., Morling N., Pokorak E., Prinz M., Salas A., Schneider P.M. (2014). DNA Commission of the International Society for Forensic Genetics: Revised and extended guidelines for mitochondrial DNA typing. Forensic Sci. Int. Genet..

[113] Sekiguchi K., Kasai K., Levin B.C. (2003). Inter- and intragenerational transmission of a human mitochondrial DNA heteroplasmy among 13 maternally-related individuals and differences between and within tissues in two family members. Mitochondrion.

[114] Tsai L.C., Lin C.Y., Lee J.C., Chang J.G., Linacre A., Goodwin W. (2001). Sequence polymorphism of mitochondrial D-loop DNA in the Taiwanese Han population. Forensic Sci. Int..

[115] Guardado-Estrada M., Juarez-Torres E., Medina-Martinez I., Wegier A., Macías A., Gomez G., Cruz-Talonia F., Roman-Bassaure E., Piñero D., Kofman-Alfaro S. (2009). A great diversity of Amerindian mitochondrial DNA ancestry is present in the Mexican mestizo population. J. Hum. Genet..

[116] Nishimaki Y., Sato K., Fang L., Ma M., Hasekura H., Boettcher B. (1999). Sequence polymorphism in the mtDNA HV1 region in Japanese and Chinese. Leg. Med..

[117] Alvarez J.C., Johnson D.L., Lorente J.A., Martinez-Espin E., Martinez-Gonzalez L.J., Allard M., Wilson M.R., Budowle B. (2007). Characterization of human control region sequences for Spanish individuals in a forensic mtDNA data set. Leg. Med..

[118] Prieto L., Zimmermann B., Goios A., Rodriguez-Monge A., Paneto G.G., Alves C., Alonso A., Fridman C., Cardoso S., Lima G. (2011). The GHEP-EMPOP collaboration on mtDNA population data—A new resource for forensic casework. Forensic Sci. Int. Genet..

[119] Parson W., Dür A. (2007). EMPOP—A forensic mtDNA database. Forensic Sci. Int. Genet..

[120] Woerner A.E., Ambers A., Wendt F.R., King J.L., Moura-Neto R.S., Silva R., Budowle B. (2018). Evaluation of the precision ID mtDNA whole genome panel on two massively parallel sequencing systems. Forensic Sci. Int. Genet..

[121] Wood M.R., Sturk-Andreaggi K., Ring J.D., Huber N., Bodner M., Crawford M.H., Parson W., Marshall C. (2019). Resolving mitochondrial haplogroups B2 and B4 with next-generation mitogenome sequencing to distinguish Native American from Asian haplotypes. Forensic Sci. Int. Genet..

[122] Kayser M., de Knijff P. (2011). Improving human forensics through advances in genetics, genomics and molecular biology. Nat. Rev. Genet..

[123] Tozzo P., Politi C., Delicati A., Gabbin A., Caenazzo L. (2021). External visible characteristics prediction through SNPs analysis in the forensic setting: A review. FBL.

[124] Walsh S., Liu F., Ballantyne K.N., van Oven M., Lao O., Kayser M. (2011). IrisPlex: A sensitive DNA tool for accurate prediction of blue and brown eye colour in the absence of ancestry information. Forenic Sci. Int. Genet..

[125] Walsh S., Chaitanya L., Clarisse L., Wirken L., Draus-Barini J., Kovatsi L., Maeda H., Ishikawa T., Sijen T., de Knijff P. (2014). Developmental validation of the HIrisPlex system: DNA-based eye and hair colour prediction for forensic and anthropological usage. Forensic Sci. Int. Genet..

[126] Peng F., Zhu G., Hysi P.G., Eller R.J., Chen Y., Li Y., Hamer M.A., Zeng C., Hopkins R.L., Jacobus C.L. (2019). Genome-Wide Association Studies Identify Multiple Genetic Loci Influencing Eyebrow Color Variation in Europeans. J. Investig. Dermatol..

[127] Hernando B., Ibañez M.V., Deserio-Cuesta J.A., Soria-Navarro R., Vilar-Sastre I., Martinez-Cadenas C. (2018). Genetic determinants of freckle occurrence in the Spanish population: Towards ephelides prediction from human DNA samples. Forensic Sci. Int. Genet..

[128] Pośpiech E., Karłowska-Pik J., Marcińska M., Abidi S., Andersen J.D., Berge M.V.D., Carracedo Á., Eduardoff M., Freire-Aradas A., Morling N. (2015). Evaluation of the predictive capacity of DNA variants associated with straight hair in Europeans. Forensic Sci. Int. Genet..

[129] Marcińska M., Pośpiech E., Abidi S., Andersen J.D., van den Berge M., Carracedo Á., Eduardoff M., Marczakiewicz-Lustig A., Morling N., Sijen T. (2015). Evaluation of DNA variants associated with androgenetic alopecia and their potential to predict male pattern baldness. PLoS ONE.

[130] Liu F., Zhong K., Jing X., Uitterlinden A.G., Hendriks A.E.J., Drop S.L.S., Kayser M. (2019). Update on the predictability of tall stature from DNA markers in Europeans. Forensic Sci. Int. Genet..

[131] Ambroa-Conde A., Casares de Cal M.A., Gómez-Tato A., Robinson O., Mosquera-Miguel A., de la Puente M., Ruiz-Ramírez J., Phillips C., Lareu M.V., Freire-Aradas A. (2024). Inference of tobacco and alcohol consumption habits from DNA methylation analysis of blood. Forensic Sci. Int. Genet..

[132] Dixon R., Egan S., Hughes S., Chapman B. (2023). The Sexome—A proof of concept study into microbial transfer between heterosexual couples after sexual intercourse. Forensic Sci. Int..

[133] Kayser M., Branicki W., Parson W., Phillips C. (2023). Recent advances in Forensic DNA Phenotyping of appearance, ancestry and age. Forensic Sci. Int. Genet..

[134] Koch C.M., Wagner W. (2011). Epigenetic-aging-signature to determine age in different tissues. Aging.

[135] Cho S., Jung S.E., Hong S.R., Lee E.H., Lee J.H., Lee S.D., Lee H.Y. (2017). Independent validation of DNA-based approaches for age prediction in blood. Forensic Sci. Int. Genet..

[136] Freire-Aradas A., Phillips C., Girón-Santamaría L., Mosquera-Miguel A., Gómez-Tato A., Casares de Cal M., Álvarez-Dios J., Lareu M.V. (2018). Tracking age-correlated DNA methylation markers in the young. Forensic Sci. Int. Genet..

[137] McEwen L.M., O’Donnell K.J., McGill M.G., Edgar R.D., Jones M.J., MacIsaac J.L., Lin D.T.S., Ramadori K., Morin A., Gladish N. (2020). The PedBE clock accurately estimates DNA methylation age in pediatric buccal cells. Proc. Natl. Acad. Sci. USA.

[138] Koop B.E., Reckert A., Becker J., Han Y., Wagner W., Ritz-Timme S. (2020). Epigenetic clocks may come out of rhythm-implications for the estimation of chronological age in forensic casework. Int. J. Leg. Med..

[139] Freire-Aradas A., Girón-Santamaría L., Mosquera-Miguel A., Ambroa-Conde A., Phillips C., Casares de Cal M., Gómez-Tato A., Álvarez-Dios J., Pospiech E., Aliferi A. (2022). A common epigenetic clock from childhood to old age. Forensic Sci. Int. Genet..

[140] Phipps M., Petricevic S. (2007). The tendency of individuals to transfer DNA to handled items. Forensic Sci. Int..

[141] Cavanaugh S.E., Bathrick A.S. (2018). Direct PCR amplification of forensic touch and other challenging DNA samples: A review. Forensic Sci. Int. Genet..

[142] Tozzo P., Mazzobel E., Marcante B., Delicati A., Caenazzo L. (2022). Touch DNA Sampling Methods: Efficacy Evaluation and Systematic Review. Int. J. Mol. Sci..

[143] Ladd C., Adamowicz M.S., Bourke M.T., Scherczinger C.A., Lee H.C. (1999). A systematic analysis of secondary DNA transfer. J. Forensic Sci..

[144] van den Berge M., Ozcanhan G., Zijlstra S., Lindenbergh A., Sijen T. (2016). Prevalence of human cell material: DNA and RNA profiling of public and private objects and after activity scenarios. Forensic Sci. Int. Genet..

[145] Alessandrini F., Cecati M., Pesaresi M., Turchi C., Carle F., Tagliabracci A. (2003). Fingerprints as evidence for a genetic profile: Morphological study on fingerprints and analysis of exogenous and individual factors affecting DNA typing. J. Forensic Sci..

[146] Burrill J., Daniel B., Frascione N. (2019). A review of trace "Touch DNA" deposits: Variability factors and an exploration of cellular composition. Forensic Sci. Int. Genet..

[147] Bonsu D.O.M., Higgins D., Austin J.J. (2020). Forensic touch DNA recovery from metal surfaces—A review. Sci. Justice J. Forensic Sci. Soc..

[148] Martin B., Linacre A. (2020). Direct PCR: A review of use and limitations. Sci. Justice J. Forensic Sci. Soc..

[149] Federal Bureau of Investigation (FBI) (2020). Quality Assurance Standards for Forensic DNA Testing Laboratories. U.S. Department of Justice. https://www.swgdam.org/_files/ugd/4344b0_d73afdd0007c4ed6a0e7e2ffbd6c4eb8.pdf.

[150] van Oorschot R.A.H., Phelan D.G., Furlong S., Scarfo G.M., Holding N.L., Cummins M.J. (2003). Are you collecting all the available DNA from touched objects?. Int. Congr. Ser..

[151] Harbison S., Marita F., Bushell D. (2008). An analysis of the success rate of 908 trace DNA samples submitted to the Crime Sample Database Unit in New Zealand. Aust. J. Forensic Sci..

[152] Quinones I., Daniel B. (2012). Cell free DNA as a component of forensic evidence recovered from touched surfaces. Forensic Sci. Int. Genet..

[153] Ottens R., Templeton J., Paradiso V., Taylor D., Abarno D., Linacre A. (2013). Application of direct PCR in forensic casework. Forensic Sci. Int. Genet. Suppl. Ser..

[154] Buckingham A.K., Harvey M.L., van Oorschot R.A.H. (2016). The origin of unknown source DNA from touched objects. Forensic Sci. Int. Genet..

[155] Lowe A., Murray C., Whitaker J., Tully G., Gill P. (2002). The propensity of individuals to deposit DNA and secondary transfer of low level DNA from individuals to inert surfaces. Forensic Sci. Int..

[156] (2016). Minimizing the Risk of Human DNA Contamination in Products Used to Collect, Store and Analyze Biological Material for Forensic Purposes.

[157] Wilson-Wilde L., Yakovchyts D., Neville S., Maynard P., Gunn P. (2017). Investigation into ethylene oxide treatment and residuals on DNA and downstream DNA analysis. Sci. Justice.

[158] European DNA Profiling Group (EDNAP), European Network of Forensic Science Institutes (ENFSI) (2023). Guideline for DNA Contamination Minimization in DNA Laboratories (ENFSI DNA-GDL-003).

[159] Vaněk D., Sasková L., Votrubová J. (2017). Does the new ISO 18385:2016 standard for forensic DNA-grade products need a revision?. Forensic Sci. Int. Genet. Suppl. Ser..

[160] Dowdeswell T.L. (2022). Forensic genetic genealogy: A profile of cases solved. Forensic Sci. Int. Genet..

[161] Glynn C.L. (2022). Bridging Disciplines to Form a New One: The Emergence of Forensic Genetic Genealogy. Genes.

[162] Kling D., Phillips C., Kennett D., Tillmar A. (2021). Investigative genetic genealogy: Current methods, knowledge and practice. Forensic Sci. Int. Genet..

[163] Wang Z., Fang Y., Liu Z., Hao N., Zhang H.H., Sun X., Que J., Ding H. (2024). Adapting nanopore sequencing basecalling models for modification detection via incremental learning and anomaly detection. Nat. Commun..

[164] Greytak E.M., Moore C., Armentrout S.L. (2019). Genetic genealogy for cold case and active investigations. Forensic Sci. Int..

[165] García Ó. (2021). Genealogía forense. Implicaciones sociales, éticas, legales y científicas. Rev. Española Med. Leg..

[166] Katsanis S.H. (2020). Pedigrees and Perpetrators: Uses of DNA and Genealogy in Forensic Investigations. Annu. Rev. Genom. Hum. Genet..

[167] Kennett D. (2019). Using genetic genealogy databases in missing persons cases and to develop suspect leads in violent crimes. Forensic Sci. Int..

[168] Wickenheiser R.A. (2019). Forensic genealogy, bioethics and the Golden State Killer case. Forensic Sci. Int. Synerg..

[169] Tillmar A., Sturk-Andreaggi K., Daniels-Higginbotham J., Thomas J.T., Marshall C. (2021). The FORCE Panel: An All-in-One SNP Marker Set for Confirming Investigative Genetic Genealogy Leads and for General Forensic Applications. Genes.

[170] Minervino A.C., Silva Júnior R.C., Corte-Real F. (2024). Advancing justice: The impact of Brazil’s convict genetic profile identification project after 5 years. Sci. Justice.

[171] Bowman Z., Daniel R., Gerostamoulos D., Woodford N., Hartman D. (2022). Rapid DNA from a disaster victim identification perspective: Is it a game changer?. Forensic Sci. Int. Genet..

[172] Turingan R.S., Vasantgadkar S., Palombo L., Hogan C., Jiang H., Tan E., Selden R.F. (2016). Rapid DNA analysis for automated processing and interpretation of low DNA content samples. Investig. Genet..

[173] Foley M.M. (2023). Rapid DNA Profile Development with Applied Biosystems RapidHIT™ ID System. Methods Mol. Biol..

[174] Turingan R.S., Tan E., Jiang H., Brown J., Estari Y., Krautz-Peterson G., Selden R.F. (2020). Developmental Validation of the ANDE 6C System for Rapid DNA Analysis of Forensic Casework and DVI Samples. J. Forensic Sci..

[175] Gupta G.Y., Gupta Y.A. (2024). From Genes to Justice: Ethical Dilemmas and Scientific Advancements in DNA Profiling for Legal Cases. Int. J. Law Manag. Humanit..

[176] Ogden R., Dawnay N., McEwing R. (2009). Wildlife DNA Forensics—Bridging the Gap Between Conservation Genetics and Law Enforcement. Endanger. Species Res..

[177] Moore M., Baker B., Bauman T., Curtis M., Espinoza E., Ferrell C., Frankham G., Frazier K., Giles J., Hawk D. (2021). The Society for Wildlife Forensic Science standards and guidelines. Forensic Sci. Int. Anim. Environ..

[178] Johnson R.N., Wilson-Wilde L., Linacre A. (2014). Current and future directions of DNA in wildlife forensic science. Forensic Sci. Int. Genet..

[179] Parson W., Pegoraro K., Niederstätter H., Föger M., Steinlechner M. (2000). Species identification by means of the cytochrome b gene. Int. J. Leg. Med..

[180] Parson W., Brandstätter A., Alonso A., Brandt N., Brinkmann B., Carracedo A., Corach D., Froment O., Furac I., Grzybowski T. (2004). The EDNAP mitochondrial DNA population database (EMPOP) collaborative exercises: Organisation, results and perspectives. Forensic Sci. Int..

[181] Vasiljevic N., Lim M., Humble E., Seah A., Kratzer A., Morf N.V., Prost S., Ogden R. (2021). Developmental validation of Oxford Nanopore Technology MinION sequence data and the NGSpeciesID bioinformatic pipeline for forensic genetic species identification. Forensic Sci. Int. Genet..

[182] Ogden R., Vasiljevic N., Prost S. (2021). Nanopore sequencing in non-human forensic genetics. Emerg. Top. Life Sci..

[183] Ogden R., Linacre A. (2015). Wildlife forensic science: A review of genetic geographic origin assignment. Forensic Sci. Int. Genet..

[184] Berger B., Berger C., Hecht W., Hellmann A., Rohleder U., Schleenbecker U., Parson W. (2014). Validation of two canine STR multiplex-assays following the ISFG recommendations for non-human DNA analysis. Forensic Sci. Int. Genet..

[185] Morf N., Kopps A., Nater A., Lendvay B., Vasiljevic N., Webster L., Fautley R., Ogden R., Kratzer A. (2021). STRoe deer: A validated forensic STR profiling system for the European roe deer (Capreolus Capreolus). Forensic Sci. Int. Anim. Environ..

[186] Dormontt E.E., Jardine D.I., van Dijk K.J., Dunker B.F., Dixon R.R.M., Hipkins V.D., Tobe S., Linacre A., Lowe A.J. (2020). Forensic validation of a SNP and INDEL panel for individualisation of timber from bigleaf maple (*Acer macrophyllum* Pursch). Forensic Sci. Int. Genet..

[187] Woodcock L., Gooch J., Wolff K., Daniel B., Frascione N. (2023). Fingermarks in wildlife forensics: A review. Forensic Sci. Int..

[188] Baxter J.R., Kotze A., de Bruyn M., Matlou K., Labuschagne K., Mwale M. (2024). DNA barcoding of southern African mammal species and construction of a reference library for forensic application. Genome.

[189] (2024). Standard for the Selection and Evaluation of GenBank® Results for Taxonomic Assignment of Wildlife, 1st ed.

[190] Frankham G.J., Ogden R., Baker B.W., Ewart K.M., Johnson R.N., Kuiper I., Lindquist C.D., Moore M.K., Ndiaye A. (2025). Webster LMI: Standards in wildlife forensic science, with a focus on non-human DNA analysis. Anim. Genet..

[191] García-Aceves M.E., Romero Rentería O., Díaz-Navarro X.X., Rangel-Villalobos H. (2018). Paternity tests in Mexico: Results obtained in 3005 cases. J. Forensic Leg. Med..

[192] Turrina S., Bortoletto E., Giannini G., De Leo D. (2021). Monozygotic twins: Identical or distinguishable for science and law?. Med. Sci. law.

[193] Chen A., Tao R., Li C., Zhang S. (2022). Investigation on the genetic-inconsistent paternity cases using the MiSeq FGx system. Forensic Sci. Res..

[194] Gettings K.B., Bodner M., Borsuk L.A., King J.L., Ballard D., Parson W., Benschop C.C.G., Børsting C., Budowle B., Butler J.M. (2024). Recommendations of the DNA Commission of the International Society for Forensic Genetics (ISFG) on short tandem repeat sequence nomenclature. Forensic Sci. Int. Genet..

[195] Churchill J.D., Schmedes S.E., King J.L., Budowle B. (2016). Evaluation of the Illumina (R) beta version ForenSeqTMDNA signature prep kit for use in genetic profiling. Forensic Sci. Int. Genet..

[196] Wang Z., Zhou D., Wang H., Jia Z., Liu J., Qian X., Li C., Hou Y. (2017). Massively parallel sequencing of 32 forensic markers using the Precision ID GlobalFiler™ NGS STR Panel and the Ion PGM™ System. Forensic Sci. Int. Genet..

[197] Montano E.A., Bush J.M., Garver A.M., Larijani M.M., Wiechman S.M., Baker C.H., Wilson M.R., Guerrieri R.A., Benzinger E.A., Gehres D.N. (2018). Optimization of the Promega PowerSeq™ Auto/Y system for efficient integration within a forensic DNA laboratory. Forensic Sci. Int. Genet..

[198] Riman S., Iyer H., Borsuk L.A., Vallone P.M. (2020). Understanding the characteristics of sequence-based single-source DNA profiles. Forensic Sci. Int. Genet..

[199] Connell J.R., Benton M.C., Lea R.A., Sutherland H.G., Haupt L.M., Wright K.M., Griffiths L.R. (2022). Evaluating the suitability of current mitochondrial DNA interpretation guidelines for multigenerational whole mitochondrial genome comparisons. J. Forensic Sci..

[200] Liu G., Zheng Y., Wu Q., Feng T., Xia Y., Chen D., Ren L., Bai X., Li Q., Chen D. (2023). Assessment of ForenSeq mtDNA Whole Genome Kit for forensic application. Int. J. Leg. Med..

[201] Verogen, Inc ForenSeq Imagen Kit Reference Guide [Internet]. San Diego, CA: Verogen; 2022 [Revised 15th April 2025]. https://verogen.com/wp-content/uploads/2022/08/forenseq-imagen-reference-guide-PCR1-vd2022008-a.pdf.

[202] Garneau J.E., Dupuis M.-È., Villion M., Romero D.A., Barrangou R., Boyaval P., Fremaux C., Horvath P., Magadán A.H., Moineau S. (2010). The CRISPR/Cas bacterial immune system cleaves bacteriophage and plasmid DNA. Nature.

[203] Dash H.R., Arora M. (2022). CRISPR-CasB technology in forensic DNA analysis: Challenges and solutions. Appl. Microbiol. Biotechnol..

[204] Sobral A.F., Dinis-Oliveira R.J., Barbosa D.J. (2025). CRISPR-Cas technology in forensic investigations: Principles, applications, and ethical considerations. Forensic Sci. Int. Genet..

[205] Barash M., McNevin D., Fedorenko V., Giverts P. (2024). Machine learning applications in forensic DNA profiling: A critical review. Forensic Sci. Int. Genet..

[206] Afkanpour M., Momeni M., Tabrizi A.A., Tabesh H. (2024). A haplogroup-based methodology for assigning individuals to geographical regions using Y-STR data. Forensic Sci. Int..

[207] Sessa F., Esposito M., Cocimano G., Sablone S., Karaboue M.A.A., Chisari M., Albano D.G., Salerno M. (2024). Artificial Intelligence and Forensic Genetics: Current Applications and Future Perspectives. Appl. Sci..

